# Architecture of systems affecting disease trajectories in a conflict zone: A community-centered systems inquiry in North Gaza

**DOI:** 10.1371/journal.pgph.0004450

**Published:** 2025-09-10

**Authors:** Yahya Shaikh, Mohammed O. A. Hamouda, Shatha Elnakib

**Affiliations:** 1 Independent Physician and Researcher, Baltimore, Maryland, United States of America; 2 General Pediatrics, Al-Rantissi Naser Paediatric Hospital, Gaza Strip, Palestine; 3 Faculty of Medicine, Islamic University of Gaza, Gaza Strip, Palestine; 4 Department of International Health, Center for Humanitarian Health, Johns Hopkins Bloomberg School of Public Health, Baltimore, Maryland, United States of America; Royal Infirmary of Edinburgh, UNITED KINGDOM OF GREAT BRITAIN AND NORTHERN IRELAND

## Abstract

Humanitarian crises, particularly in conflict zones, create cascading disruptions that impact every aspect of daily life, including health and disease outcomes. While international humanitarian frameworks categorize these crises into discrete operational clusters, affected populations experience them as interwoven, systemic failures. This study examines how conflict-induced disruptions transform a preventable and typically self-limiting disease—Hepatitis A—into a fatal outcome. Using a systems approach, we seek to characterize the architecture of interconnected disruptions leading to preventable deaths. This study employed the FAIR (Fairness, Agency, Inclusion, and Representation) Framework, a participatory methodology centering community epistemes, to analyze four pediatric cases of Hepatitis A that progressed to fulminant liver failure. Data were obtained through interviews with healthcare providers, caregivers, and community members, supplemented by medical chart reviews. A network-based Architecture of Systems (AoS) map was constructed to visualize interconnections between war-induced systemic disruptions and health outcomes. Network analysis identified key nodes and pathways within the systems map. The findings of this study reveal a complex system of war-driven factors including displacement, destruction of healthcare infrastructure, water scarcity, food deprivation, and fuel blockades that collectively reshaped disease trajectories. Network analysis of the AoS map identified 138 nodes and 231 edges, generating 34,458 pathways linking conflict-related disruptions to health outcomes. Women’s health emerged as a central mediator, with 97% of pathways intersecting with 25 key nodes including women’s roles in caregiving, resource acquisition, and psychological stability. The lack of access to food and clean water, combined with the destruction of healthcare facilities and restrictions on medical evacuation, created conditions where preventable, self-limiting diseases become fatal. This study highlights how conflict restructures health determinants, turning survival strategies into pathways of increasing morbidity and mortality. It also underscores the need for a systems-based humanitarian response that considers the intersecting pathways driving outcomes in crisis settings.

## Introduction

Humanitarian crises, particularly in times of conflict, are rarely experienced by affected populations as isolated events or discrete challenges. Instead, they manifest as interlinked, cascading systems of disruption that reshape every aspect of daily life, force movement, restrict choices, dictate behaviors, and ultimately worsen health, disease, and mortality [1–3]. While the humanitarian sector often deconstructs these crises into operational clusters such as food security, healthcare, or shelter to facilitate aid [4–6], this fragmented perspective risks overlooking the lived experiences of those enduring the crisis [3,7]. For affected populations, these sectors are inseparable components of a deteriorating or adversely reconstructed system. The failure to routinely characterize this system means that representations of the affected community’s realities are incongruent with their lived experiences [8–13]. This, in effect, results in silencing the community’s voice [14], upholding a system that has adverse impacts on the population [11,15–17], and constitutes epistemic violence. Epistemic violence occurs when the experience of affected populations is reframed by external actors in ways that distort or diminish their reality [14,18–22]. In humanitarian crises, this is particularly problematic, as aid frameworks designed for logistical clarity often fail to capture the systemic interdependence of events as they are experienced by the community [7]. For instance, the bombing of residential areas may lead to displacement, which, coupled with restrictions on food and water transport, creates conditions for malnutrition and disease outbreaks. These interconnected phenomena are not experienced as separate “sectors” but as a coherent, worsening system of survival challenges.

Emerging research emphasizes the need for a systems perspective in understanding and responding to humanitarian crises [3]. By examining humanitarian crises as systems of effects, researchers and practitioners can more accurately represent the complex realities of affected populations, ensuring that interventions address individual factors along with the relationships between them [7,23]. This perspective is essential to ensure that a population attempting to survive a humanitarian crises is not additionally violated by epistemic violence in the name of humanitarian relief [22].

Hepatitis A provides an important lens for applying this systems perspective because it illustrates how interconnected factors during humanitarian crises can transform a preventable, self-limiting disease into a fatal outcome. Studies have shown that conflict-induced conditions such as overcrowding, lack of sanitation, and destruction of water systems create environments where diseases with fecal-oral transmission, like Hepatitis A, spread rapidly [24,25]. Simultaneously, restricted access to healthcare, exacerbated by destroyed infrastructure and medical supply shortages, limits timely diagnosis and treatment, allowing complications to escalate [25]. These interacting factors arising from systemic disruptions highlight the need to view health outcomes not as isolated clinical phenomena but as products of broader socio-political and infrastructural destruction. In this article, we characterize the system leading to child deaths from Hepatitis A, a rare complication of a preventable disease [26]. Using the FAIR (Fairness, Agency, Inclusion, and Representation) Framework [27], a participatory research methodology to center community epistemes, we ask and attempt to answer the question: What is the architecture of the system of interconnected disruptions during a humanitarian crisis that transforms a preventable disease (like Hepatitis A) into a fatal outcome? This inquiry seeks to uncover pathways through which socio-political and infrastructural destruction amplify risks, restrict choices, and shape survival strategies, ultimately contributing to adverse health outcomes. By centering the lived experiences of the affected community, this study attempts to uncover mechanisms of morbidity and mortality when civilian populations are impacted by military operations.

## Methods

### Statement of ethics

Primary data collection for this study, including construction of the architecture of systems map was initially conducted as part of an environmental assessment for humanitarian purposes. The study protocol to conduct subsequent analysis (e.g., network analysis) on collected data was reviewed by Johns Hopkins University IRB (IRB No: 31883) and received an exemption as it involved the use of existing secondary data. To ensure confidentiality and security, no individual identifiers were recorded. Verbal informed consent was obtained from all participants prior to participation, further safeguarding privacy amid security risks. Discussion groups were conducted by the first two authors. While the original study was conducted as part of a needs assessment, data was accessed for the purpose of this study on 2/2/2025.

### Theoretical framework

This study employs a participatory, systems-based approach to understanding humanitarian crises, particularly focused on how conflict reshapes the determinants of health in affected populations. The theoretical foundation draws from three related frameworks that together provide a comprehensive lens for examining disease trajectories in conflict zones. Applying systems theory in humanitarian contexts allows us to conceptualize conflict zones as complex adaptive systems where disruptions cascade through interconnected social, economic, and physical infrastructures. This perspective aligns with emerging research emphasizing the need to understand humanitarian crises as systems of effects rather than isolated challenges. Complementing this, a social determinants of health lens recognizes that health outcomes are shaped by broader social, political, and environmental factors. In conflict settings, these determinants are drastically transformed, creating new pathways to morbidity and mortality that cannot be understood through traditional clinical perspectives alone. Central to our methodology is participatory epistemology, which recognizes that affected communities possess critical knowledge about the systems that shape their lives. The study privileges local knowledge systems and community epistemes as essential to understanding the complex realities of conflict zones. This integrated theoretical perspective guides our investigation of how war-driven disruptions transform a preventable disease into a fatal outcome, focusing on the architecture of the system rather than isolated factors. To operationalize these theoretical commitments in this study we employ the FAIR Framework a participatory approach to knowledge production that prioritizes the perspectives and epistemologies of communities, and recognizes the agency of communities in representing and interpreting their own experiences [22,27]. It engages community members to convey the context of their lived experience and to translate their insights into systems of understanding that inform external researchers and policymakers. This approach bridges the gap between community epistemologies and dominant systems of knowledge, creating outputs that are more accurate, equitable, and aligned with the lived experiences of those on the margins [27].

### Study design and context

This study was conducted in the period spanning June 27^th^, 2024 to July 25^th^, 2024 in Gaza during an extreme humanitarian crisis amid an ongoing military campaign by Israel that began in October 2023. As of the time of this study, Israeli forces had destroyed most of the hospitals in Gaza, with only 13 of Gaza’s 36 hospitals remaining partially functional. North Gaza, the site of this study, had three partially functioning hospitals, one of which was a pediatric hospital (Kamal Adwan Hospital) serving a remaining population of about 75,000 people [28,29]. Since the onset of this war, North Gaza has faced a “siege within a siege,” with severe restrictions on food and medical supplies. By March-April 2024, these restrictions resulted in widespread hunger and malnutrition, with the civilian population reaching Phase 4 (Emergency) of the Integrated Food Security Phase Classification [30]. Many residents survived by consuming animal feed and foraged weeds.

This study aimed to examine the systemic factors contributing to deaths from Hepatitis A, a typically preventable disease, by focusing on four pediatric patient cases who presented to hospitals in North Gaza between March and April 2024. The study used a participatory methodology to engage healthcare providers, families, and caregivers to uncover systemic barriers that contributed to these fatalities.

### Reflexivity and positionality of the researcher

The research team brought complementary perspectives and experiences to this study, with direct connections to the cases under investigation. The first author (YS) was deployed to North Gaza as a physician during the conflict, providing clinical care to affected populations while simultaneously documenting systemic barriers to healthcare. This dual role as both healthcare provider and researcher offered unique insights into the immediate challenges facing patients and providers, while also requiring careful reflection on the emotional and ethical complexities of conducting research amid ongoing violence.

MOAH, a Palestinian pediatrician, treated the study’s patients at Al-Rantissi Naser Pediatric Hospital until the facility was destroyed by Israeli strikes, then continued caring for children in the pediatric intensive care unit (ICU) at Kamal Adwan Hospital. Providing care to patients in the midst of destruction, displacement, and acute resource shortages made him both an affected clinician and a researcher of Gaza’s systemic health failures. This dual role offered granular insight into disease trajectories and real-time treatment barriers, but also demanded constant reflexivity when analyzing cases he had personally managed. Some safeguards mitigated his context of a researcher examining the very context that affected him: (i) a participatory approach in which community members co-shaped coding, clustering, and systems mapping; (ii) analytic triangulation with co-authors, followed by iterative member-checking discussions, with divergent interpretations among co-authors resolved by privileging Gaza-based community voices; and (iii) regular peer-support debriefs with co-authors, providing a confidential space to process moral distress and keep interpretations balanced.

SE, a public health scholar, contributed expertise in humanitarian systems while supporting analysis and interpretation from outside the immediate conflict zone. The team acknowledged these positional differences and prioritized community insights through iterative dialogue with participants, who critiqued and validated interpretations of their experiences.

By employing the FAIR Framework as a methodological approach, the team sought to center the knowledge and experiences of those directly affected by the conflict, recognizing that community members and local healthcare providers hold essential expertise about the systems shaping health outcomes. To mitigate potential biases, the team employed participatory validation, where community members and healthcare providers reviewed and refined the systems map. Reflexivity was embedded through iterative discussions with participants to challenge assumptions and ensure interpretations aligned with community perspectives. Researchers engaged in regular reflexive discussions about the tensions between documenting suffering and responding to immediate needs, and how their positions influenced data collection and interpretation. Researchers acknowledged their positional power as external actors and prioritized local voices by structuring interviews as collaborative dialogues rather than extractive inquiries.

Throughout the research process, the team acknowledged that complete neutrality is impossible when documenting human suffering during armed conflict, particularly when researchers have been directly involved in patient care and instead aimed for transparency about their positions and perspectives.

### Participants

The study focused on four pediatric patients who experienced disease progression to fulminant liver failure and death. Inclusion criteria for participants in the study were as follows:

- Healthcare providers involved in the care of these four patients.
- Family members and caregivers who provided additional insights into patient health journeys.
- Community members familiar with the broader socio-political context shaping access to healthcare.

Participants were excluded if they were unable to safely participate due to ongoing security threats at time of recruitment.

Given the extreme security risks and constraints, participant engagement was adapted to ensure feasibility and safety, including condensed sessions, flexible meeting times, and modified data collection methods.

### Data collection

This study employed the FAIR Framework, which consists of a multi-step process designed to center community knowledge and experiences in understanding systemic challenges. It begins with Knowledge-Bearer and Knowledge-Interpreter recruitment, identifying community members with deep, lived expertise and the ability to articulate and contextualize their community’s realities. This is followed by relationship building to establish trust, ensure mutual understanding, and create a collaborative space for exploring community knowledge. Next, in vignette development and enrichment, participants craft detailed narratives that reflect diverse lived experiences within the community, which are then analyzed to identify cause-effect pathways. These analyses led to the construction of an architecture of systems map, a visual representation of the relationships between key factors shaping systemic challenges. The map is subsequently augmented using literature and additional insights to enhance its accuracy and applicability. Each step is iterative and participatory, ensuring that outputs authentically represent the community’s epistemologies while fostering actionable insights for addressing systemic inequities.

**Fig 1:**
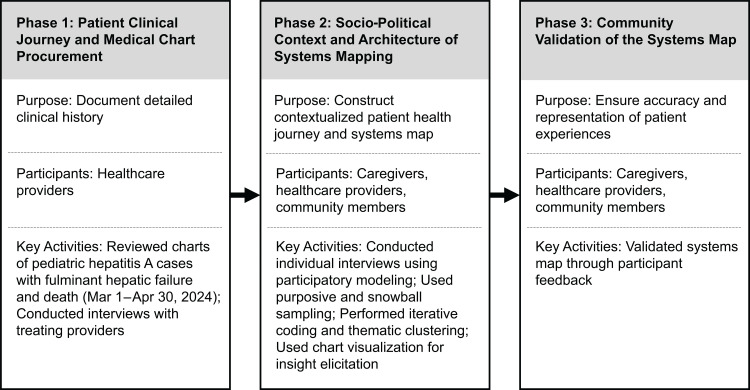
Phases of the FAIR Framework modified for contexts of war.

The methodology was adapted to the war context in Gaza and structured into three phases as given in Fig 1.

A chart visualizing causes as nodes and cause-effect relationships as edges was used in Phase 2 (see Fig 1) to engage participants using insight elicitation scripts (see S1 File) to develop an Architecture of Systems (AoS) Map. A comparison of the original FAIR Framework and the modified FAIR Framework for contexts of war is given in Table 1.

**Table 1:** Original FAIR Framework and modified FAIR framework for contexts of war.

FAIR Framework step	Key features in the original FAIR Framework	Key features in the FAIR Framework adapted for contexts of war	Reason for change in active-war context
**1. Recruit Knowledge-Bearer(s) & Knowledge-Interpreter(s)**	Recruit community elder/advocate–researcher with long experience and broad network.	Recruit treating pediatricians, caregivers, and community members sharing the patient’s context (e.g., family members, neighbors); their roles were pre-existing in connection to the cases, rather than recruited.	Examining an active war context meant a focus on knowing through experiencing. Knowledge bearers and interpreters were those clinicians, caregivers, family and community members who lived, observed, and experienced the same context as the children being examined in this study.
**2. Relationship building**	Several preliminary dialogues to establish trust, clarify goals, and ensure the collaboration would be empowering rather than extractive.	Trust and rapport were implicit in shared crisis; sessions were condensed and scheduled ad-hoc to minimize security risk.	Constant shelling, hospital evacuations and communication blackouts meant security was a priority, with minimal time for preliminary conversations. However, conducting the study within a shared context of war meant implicit trust and bond developed between researchers and researched.
**3. Session introduction, vignette development & enrichment**	Multi-session script: participants write 3–5 composite vignettes capturing diverse poverty trajectories; guided probing using the Williams–Mohammed framework and Ecosystems-of-Opportunities meta-model.	Phase 1: patient histories were the vignettes, with histories constructed through chart review plus clinically-oriented narratives of each child’s journey (history embedded in medical notes). Phase 2 (part-1): caregivers & staff extended those journeys with socio-political context.	Vignette-crafting from knowledge-bearers was not needed as medical charts containing patient histories were available from which “vignettes” could be constructed.
**4. Vignette analysis & constructing the Architecture-of-Systems map**	Sessions of open/axial/selective coding with the Interpreter; factors grouped, linked and visualized in a systems map.	Phase 2 (part-2): rather than sessions involving conversations with all community members at one time, the context of war meant an iterative approach to coding/clustering of barriers affecting the studied children: all community members could not meet at the same time in the same place; rather researchers met community members multiple times, at different times and at different places (based on security and availability) to cross-validate coding, clustering, and building of the systems map.	Analytical mechanics (coding to mapping) were retained, but how meetings were held was adapted to active conflict zone: instead of single meetings, there were multiple and iterative meetings.
**5. Augmenting the map with literature & additional data**	Post-analysis literature search for every edge in the map; citations appended; gaps filled.	Phase 3: replaced by community validation meetings where providers & caregivers verified accuracy.	Real-time validation by local actors was more achievable and directly actionable. Due to the active nature of the war, community experience and knowledge was more up-to-date than academic literature.

This study examines how an acute humanitarian crisis transforms disease trajectories. The post-October 7th period in Gaza created qualitatively different conditions moving from chronic constraints to acute system collapse, which fundamentally altered survival strategies and health determinants. For these reasons, in constructing the AoS map, we restricted systems boundaries to the period after the October 7, 2023 escalation.

### Data analysis

Once the architecture of systems diagram was constructed, network analysis was conducted to identify key nodes and their centrality measures. The analysis aimed to uncover the most connected, influential, and accessible nodes in the network. Key network metrics were calculated to understand the structure and significance of nodes within the network and included:

- Degree centrality: the number of direct connections a node has (both incoming and outgoing), normalized by the maximum possible connections in the network. This metric identified the most connected nodes, representing highly influential factors or those most affected.
- Betweenness centrality: the frequency with which a node acts as a bridge along the shortest paths between other nodes. Nodes with high betweenness centrality indicate critical bottlenecks or intermediaries where interventions may have the greatest cascading effects.
- Closeness centrality (factors most accessible within the network): the closeness a node is to all other nodes in the network, measured as the reciprocal of the average shortest path length from the node to all others. Nodes with high closeness centrality are centrally positioned and readily accessible, indicating factors that can rapidly influence or be influenced by others.

Network analysis was performed using Python version 3.11.

## Results

This study included 25 participants (7 healthcare workers; 18 community members including parents, family, and neighbors) with a mean age of 35 + /-9 years, of whom 10 (40%) were women. Using the FAIR Framework, over a span of on average 2 sessions per person, with some sessions occurring with multiple people at the same time for a total of 19 sessions, the study explored the system that shapes patient trajectories of a preventable and self-limited disease towards fatal outcomes in North Gaza. The findings reveal a complex interplay of precipitating factors and pathways driven by war-induced disruptions, highlighting the systemic nature of humanitarian crisis.

The patient journeys obtained from medical charts, providers, and caregivers (Phase 1 of the modified FAIR Framework) related to four otherwise healthy children with similar histories and clinical trajectories who presented with hepatitis A which subsequently progressed to fulminant hepatic failure and death. All four children were previously healthy, aged 7–10 years old, brought at various times between April and June 2024 to the Emergency Department of a community hospital in North Gaza after several weeks of progressively worsening symptoms that included abdominal pain, loss of appetite, nausea, vomiting, diarrhea, icterus, and jaundice. They had initial presentations to the healthcare system where they were appropriately managed conservatively for malnutrition and hepatitis A infection. After several subsequent weeks of worsening symptoms, they were finally brought to the hospital due to lethargy and changing levels of consciousness. Their social histories were significant for multiple displacements from Internally Displaced Persons (IDP) camps due to evacuation orders, after their homes were initially destroyed in air strikes by Israeli forces. They all experienced a period of severe hunger from January 2024 to April 2024, during which time they consumed animal feed, often ground and incorporated as part of the “flour” for bread. Their risk factors (i.e., poor sanitation, limited access to clean water, crowded conditions), symptoms, clinical findings, lab tests, and imaging were indicative of acute malnutrition and hepatitis A infection with hepatic encephalopathy. The siege and ongoing air strikes on civilian infrastructure restricted the ability to diagnose and monitor, treatment options, referral options, and transport to neighboring facilities for higher levels of care for all patients.

**Fig 2:**
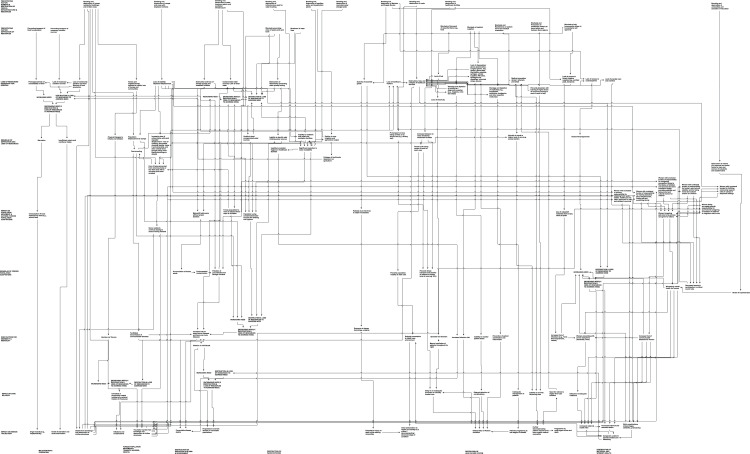
Architecture of systems diagram.

Centering community voice to identify factors shaping patient trajectories yielded 138 distinct codes, whose interrelationships were mapped in the AoS diagram (Phases 2 and 3 of the modified FAIR Framework; Fig 2). Factors that influenced other codes without being influenced themselves were considered initiating causes and positioned thematically along the X-axis of the AoS diagram. In contrast, factors that were both shaped by upstream influences and contributed to downstream effects were defined as intermediate determinants. These intermediate determinants were chronologically organized along the Y-axis, reflecting the temporal unfolding of causal pathways from initiating causes (on one end of the Y-axis) to health outcomes (on the opposite end of the Y-axis).

**Fig 3:**
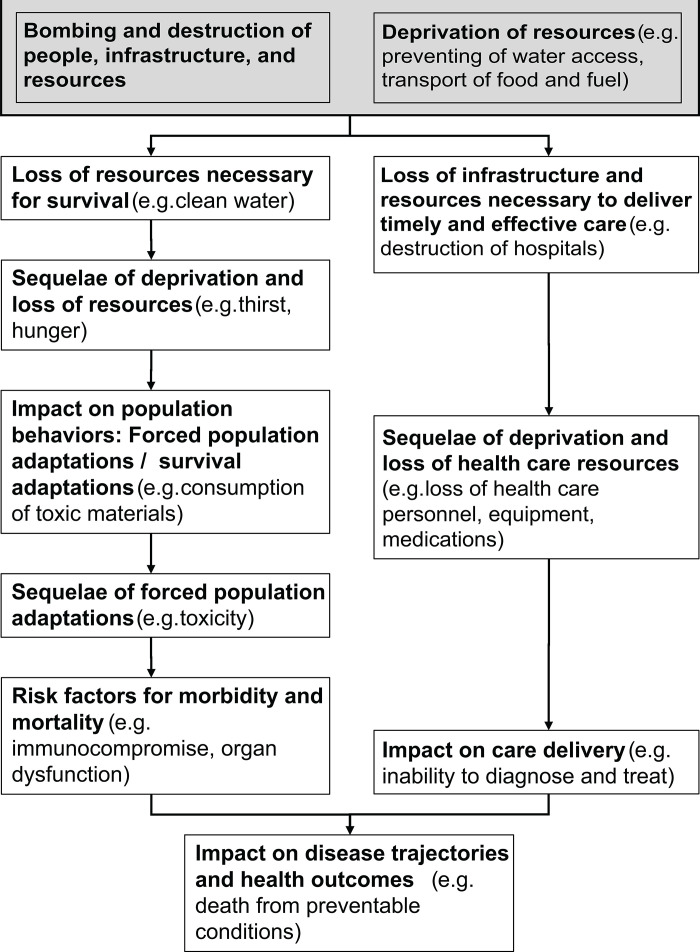
Thematic structure of cause-effect pathways.

Thematic clustering of codes that became intermediate determinants when interlinked in the AoS map produced 12 categories (Table 2), representing either the impact of bombing and deprivation on individuals (e.g., civilian deaths, starvation, maternal and neonatal morbidity and mortality, multiple displacements, and the loss of vision and hope) or effects of bombing and deprivation on population resources (destruction of community systems of food, water, and health care). Organizing the network of determinants with initiating causes positioned on one side of the Y-axis and health outcomes on the opposite side connected by intermediate factors yielded 11 strata of cascading events (Fig 3). Each stratum was associated with a distinct thematic pattern, which is reflected as labeled strata in the far-left column of the AoS diagram. Fig 3 summarizes these strata, illustrating how bombing and deprivation triggered both increased health care needs (through the loss of essential survival resources, resulting sequelae, adaptive behaviors, and their consequences) and a concurrent decline in the capacity to deliver care. These abstractions of effects (Fig 3) reveal a systemic logic that underpins the progression from destruction of an individual’s external environment to physiological collapse of their internal state of health and well-being. Specifically, pathways originating from the destruction of resources essential for survival (e.g., food, water, shelter), coupled with the deprivation of resources needed to respond to that destruction (e.g., restrictions of medical supplies, transport, healthcare personnel), bifurcate into two interrelated trajectories. The first trajectory involves a sequential loss of external survival inputs, such as access to adequate nutrition, clean water, and safe shelter, which led to harmful sequelae including hunger, dehydration, and exposure. These, in turn, trigger pathogenic survival adaptations (e.g., consumption of unsafe food or untreated water) as individuals and communities sought to endure amid deprivation. These externally imposed adaptations had direct physiological consequences, including metabolic disturbances, hepatic failure, electrolyte imbalances, and maladaptive stress responses, which are all conditions that would ordinarily be mitigated by timely medical care. The second trajectory emerging from an abstraction of effects (Fig 3) represents the simultaneous collapse of the healthcare system due to the destruction of infrastructure and blockade of critical supplies. This dual collapse of both survival resources and the healthcare response resulted in the inability of the population to address the adverse effects of pathogenic survival adaptations, ultimately leading to preventable illness and death.

**Table 2:** Thematic categories of effects (i.e., intermediate determinants) connecting initiating causes/ source nodes with outcomes.

High-Level Categories	Thematic Categories of Intermediate Determinants
**Effect of bombing and deprivation on people**	Starvation of population
	Deaths; traumatic injuries
	Maternal and neonatal morbidity and mortality
	Destruction of homes and shelter
	Loss of personal items necessary for normal life: documents and certificates, identification papers, valuables; books, medicines, clothing, household items
	Forced population movements; multiple displacements
	Loss of hope and visions of the future
**Effect of bombing and deprivation on population resources**	Destruction of food systems
	Destruction of water systems
	Destruction of healthcare system
	Destruction of education system
	Destruction of sanitation systems; contamination of environment

Thematically organizing initiating causes revealed two overarching categories (bombing and destruction of people, infrastructure, and resources; and deprivation of resources). These overarching categories encompassed 19 initiating causes that launched a system of interdependent factors (intermediate determinants), generating cascading effects, reshaping lives, and altering disease trajectories (Table 3):

**Table 3:** Initiating causes/ source nodes (primary factors initiating a system of inter-related and cascading effects).

Type of Initiating Factor	Initiating Factor
**Bombing & Destruction of People, Infrastructure & Resources**	Bombing and destruction of homes, residential buildings, and civilian areas
	Bombing and destruction of camps and areas with displaced civilians
	Bombing and destruction of water treatment plants and sanitation infrastructure
	Bombing and destruction of infrastructure used for water trucking
	Bombing and destruction of aquifers, wells, and desalination plants
	Bombing and destruction of municipal water storage tanks
	Bombing and destruction of Markets and Supply Routes
	Bombing and destruction of roads
	Bombing and destruction of healthcare facilities
	Bombing and destruction of universities and cessation of education
**Deprivation of Resources**	Preventing transport of food to population
	Preventing transport of necessary nutrition sources
	Blockade preventing entry of spare parts and tools
	Shutdown of water lines
	Blockade of transport of essential items (e.g., fuel)
	Blockade of medical supplies
	Blockade and Restrictions on patient travel and medical evacuation
	Blockade and Prevention of healthcare workers to travel within and between Gaza regions
	Blockade of aid; bureaucratic delays in humanitarian aid approval

- The bombing of residential areas displaced populations to overcrowded camps, creating environments conducive to the transmission of communicable diseases such as Hepatitis A. Repeated displacement, compounded by the bombing of camps and surrounding areas, further hindered the establishment and maintenance of safe water and sanitation facilities. These conditions facilitated the spread of waterborne diseases and other infections transmitted via the fecal-oral route, contributing to the progression of severe acute malnutrition in affected children.
- The bombing and destruction of homes, residential buildings, and civilian areas resulted in deaths, displacement, and significant disruption to family life. Women bore a disproportionate burden, taking on the responsibility for their family’s survival, stability, and serving as moral and cultural anchors for children, often at the expense of their own self-care. This heightened role increased vulnerability to communicable diseases and maternal health risks. As primary caregivers, women often faced challenges in providing effective care, leading to delays in seeking medical attention. Consequently, when individuals finally presented at healthcare facilities, their conditions were frequently advanced, critical, or decompensated, making treatment more complex and outcomes less favorable.
- The bombing and destruction of healthcare facilities forced patients to travel longer distances to access care, often under life-threatening conditions. Many patients delayed seeking care due to the risks associated with travel, leading to late-stage presentations requiring referral to tertiary care. These delays compounded the severity of illness, increasing the need for ICU-level care in already resource-constrained environments.
- The prevention of food transport to the population emerged as a critical driver of starvation, forcing individuals to consume toxic materials, such as animal feed, as a last resort. This led to organ toxicity, including hepatotoxicity, and the onset of severe acute malnutrition. Similarly, the restriction of necessary sources of nutrition resulted in insufficient caloric and nutritional intake, further exacerbating malnutrition. These pathways illustrate how systemic barriers to food access directly influenced disease progression in vulnerable populations, particularly children.
- The prevention of fuel transport to healthcare facilities disrupted essential services, including ICU operations. Hospitals were forced to rely on manual ventilation for critically ill patients, leading to inconsistent oxygen delivery, hypoxemia, and hypoxic injury. These disruptions severely compromised patient outcomes, especially for those requiring continuous monitoring and intervention.
- The embargo on medical supplies created critical shortages of laboratory reagents, medications, and equipment necessary for patient monitoring and treatment. This lack of resources resulted in delayed diagnosis and inadequate management of preventable conditions, escalating disease severity and further burdening the healthcare system.
- The denial of travel for patients to hospitals outside their area (e.g., from North Gaza to Khan Younis; or from Gaza to Jordan) prevented critical referrals and medical evacuations to facilities with higher levels of care. This lack of access to specialized treatment led to the decompensation of patients into multi-organ failure and, ultimately, death.

In considering the AoS map as a graph or network, where each factor in the AoS map is a “node” and relationships connecting nodes to each other are considered “edges”, pathways relating to women’s health were unexpectedly significant. A network analysis of the AoS map shows a graph with 138 nodes and 231 edges, resulting in 34,458 pathways (complete causal chains from initiating to terminal nodes). Notably, 25 nodes (18%) related to women’s health while 33,665 (97%) pathways connected to women’s health at some point through these nodes (see S1 File, Table A; and S2 File). When examining degree centrality of nodes, the most connected nodes (i.e., degree centrality) were: destruction, loss, and collapse of healthcare services; loss of stability; repeated displacement; significant reduction in water availability; traumatic injuries and deaths from bombings and destruction of civilian structures; and the impact of the war on women’s physical and mental health (Table 4). In the lived experience of the community, blockades, deprivation, and the bombing and destruction of people, infrastructure, and resources resulted in a lack of access to essential supplies for women’s health, including hygiene products, contraceptives, and prenatal care and nutrition. It also led to significant behavioral adaptations by communities to survive an environment of ongoing death and destruction. These behavioral adaptations included efforts necessary to fill the social and functional void left by injured, missing, and dead family members. This in turn resulted in an increasing burden on women as primary sources of survival for children and families, including becoming primarily responsible for obtaining food, water, shelter, and healthcare. It led to women shouldering a disproportionate burden of responsibility for making decisions about displacement, finding aid, and sustaining the family; for ensuring the family’s survival and stability; and for being the moral and cultural anchors for children. It placed them in roles of surrogate parenthood with a responsibility for the survival and care of children of relatives and neighbors killed in attacks. These additional burdens deprived communities of societal roles played by women (e.g., as doctors and nurses), in addition to women foregoing self-care to be primary caregivers to others, increasing their susceptibility to infections, worsening their own mental and physical health, and delaying their own treatment until they were in advanced stages of disease. These in turn led to increased maternal and neonatal morbidity and mortality, and worse outcomes in children relying on them for care.

**Table 4:** Centrality measures of Architecture of Systems map.

Node Name	Centrality Measure
Degree Centrality (Most Connected Nodes)	
Destruction, loss, and collapse of healthcare services	0.10
Significant reduction in water availability	0.06
Deaths from bombings and destruction of civilian structures	0.06
Traumatic injuries from bombings and destruction of civilian structures	0.06
Loss of stability; Repeated displacement	0.06
Women foregoing self-care to be primary care giver for others	0.05
Worsening mental health for women	0.05
Increased risk of water-borne diseases and fecal-oral route diseases	0.05
Caregivers unable to provide needed care effectively	0.04
Increased maternal health risks	0.04
Fear of being prevented from returning home or fear their land will be occupied and home annexed	0.04
Increased risk of communicable diseases for women	0.04
Bombing and destruction of homes, residential buildings, and civilian areas	0.04
Betweenness Centrality (Key Bridges or Bottlenecks)	
Destruction, loss, and collapse of healthcare services	0.05
Forces population to move to places where water is available	0.05
Women with unshared burden of responsibility for navigating occupation forces to find status of children and spouses that were arrested or killed; providing stability and being moral and cultural anchor for children	0.03
Loss of healthcare workers	0.03
Decreased women’s participation in critical social roles	0.02
Women foregoing self-care to be primary care giver for others	0.02
Caregivers unable to provide needed care effectively	0.02
Closeness Centrality (Most Accessible Nodes)	
Deterioration of Patient condition	0.11
Increased maternal and neonatal deaths	0.10
Increased maternal health risks	0.10
Progression to severe disease in vulnerable populations	0.10
Preventable disease deaths	0.10
Delay in or inadequate provision of critically needed care	0.09
Caregivers unable to provide needed care effectively	0.09
Infections and complications	0.09
Delayed presentation to health care environment	0.08
Initial presentation to health care facility is at critical/ decompensating/ advanced stage of condition	0.08
Birth complications (miscarriages, stillbirths, prematurity)	0.08
Increased risk of water-borne diseases and fecal-oral route diseases	0.07
Destruction, loss, and collapse of healthcare services	0.07

These findings illustrate that factors brought about by incidents of war interact together to create a system that restricts and shapes choices, movements, and survival strategies to the extent that preventable and self-limiting conditions become fatal. As illustrated in the AoS map, war-driven events are the foundational causes leading to a reshaping of lived experiences and resulting in a system optimized to increase morbidity and mortality.

## Discussion

### Overview of findings

This study demonstrates that anchoring findings in lived experiences can effectively uncover the system that shapes health, morbidity, disease progression, and mortality outcomes for that population. Some individual pathways revealed by the AoS map connecting military destruction of civilian infrastructure to health outcomes are supported by prior reports and studies. For example, in the AoS map (Fig 2) the community’s experience of restriction of clean water for drinking, hygiene, and healthcare and household daily use was traced to the targeting of water infrastructure by military action. This is validated by reports that document the destruction of water facilities and plants [31], wells [32,33], and reservoirs [31,34,35] by airstrikes and other military action [36], resulting in the reduction of water supply to the population in Gaza [36]. Similarly, injuries, deaths, and displacement from airstrikes, bombings, and attacks on civilian populations and infrastructure have been well documented by other reports. This study presents the novel finding that pathways that connect the destruction of a population’s civilian infrastructure to their deaths are not isolated trajectories, but interlinked and synergistic. Together, they form a system that targets the community at large while disproportionately harming women to worsen the humanitarian impact on the entire society. These adverse systems create an environment where populations die from preventable causes and a people’s strategies to survive become their pathways to death.

The study also shows that the lived experience of a population can expose the limits of humanitarian action in contexts where forces driving the crisis persist, such as ongoing bombings of civilian infrastructure, persistent blockades on essential supplies, and continuing restrictions on movement. While humanitarian organizations may work to reduce the health consequences of a crisis, this study demonstrates that improvements in health outcomes requires confronting the underlying network of factors, particularly the causal factors related to military actions directed at civilian populations and infrastructure.

### Women’s health: A central mediator of population health outcomes

This study reveals pathways that highlight the centrality of women’s health in mediating the effects of military action on civilian populations. While 25 (18%) of the 138 nodes in the AoS map directly relate to women’s health, these nodes linked to 97% of all pathways. Centrality metrics of the AoS map reveal that in Gaza almost half of the most crucial links mediating the relationship between the destruction of war and health outcomes relate to the impact of war on women. It should be noted that while women-related nodes speak directly to issues specific to women, women are also impacted by nodes that relate to both men and women (e.g., deaths and injuries that result from bombings).

The large number of pathways that intersect with nodes related to women underscores that in the lived experience of the community women’s health plays a pivotal role in mediating the effects of war on population health, particularly on children. The high degree of centrality of women-related nodes means that when women are impacted by war-related events, such as bombings and resource destruction, there are downstream effects on health outcomes for children and the entire community: attacking women’s health destroys the community’s health.

The impact of war on women as described in this study includes (amongst other impacts): the unshared burden of responsibility borne by women, caused by loss of deaths and injuries of other family members; the loss of women’s participation in critical social roles; women foregoing self-care to be primary care giver for others; worsening mental health for women; and caregivers being unable to provide needed care effectively resulting from impacts on women’s health (see Supplementary File 1, Table A; and Supplementary File 2, List of Pathways).

### War engineers and forces adverse paths to survival

The findings in this study reveal a profound and multifaceted impact of war on health outcomes, illustrating how destruction of civilian infrastructure destroys existing systems, engineering a new landscape of survival challenges for communities (Figs 2 and 3). It reveals how the effect of military action on civilian populations directly (e.g., bombing residential areas) or indirectly (e.g., destruction of water and sanitation facilities) imposes a hostile system of influences that forces communities to make choices outside the context of a functioning societal framework. Such impacts of war on civilians fundamentally reshapes the determinants of health by creating circumstances where the very actions taken for survival become direct pathways to illness and death (like eating substitute foodstuffs in the absence of safe options). This indicates that war in Gaza and potentially other regions does not merely interrupt established systems; it generates a series of forced decisions that actively undermines community health. By removing conventional choices and introducing new, perilous alternatives as survival adaptations, this engineered context escalates vulnerability, establishing an entirely different set of hazards compared to those seen in natural or accidental crises.

The pathways documented in this study demonstrate how war destroys the structures of civilian life and how that destruction enters and is reproduced within the human body (Figs 2 and 3). They show that war is embodied: war in the outside world becomes a war within the body. As individuals are forced into adaptive behaviors by loss of external resources the body takes on those external ruptures, internalizing scarcity, destruction, and collapse. The population’s forced adaptations, while necessary for survival, introduce physiological disturbances that manifest as organ failure, malnutrition, and immune disintegration. In the absence of functioning healthcare systems, these internal wars are not resolved but accelerate toward death. This alignment between external destruction and internal deterioration highlights how conflict directly causes death while simultaneously constructing a bio-political system where living itself becomes pathogenic. It reaffirms the necessity of seeing war’s health consequences not as collateral but as structured, systemic, and embodied. Interventions that fail to account for this internalization of harm may overlook the full extent of war’s impact on the body and life itself.

An additional insight emerges when examining the initiating nodes of military actions documented in our study (Table 3) in relation to established emergency management frameworks. The United States Federal Emergency Management Agency defines community lifelines as “the most fundamental services in the community that, when stabilized, enable all other aspects of society to function” [37]. When we consider the infrastructure that community members reported as being bombed and destroyed (Table 3), these align remarkably with the community lifelines that emergency management professionals recognize as essential for societal functioning (e.g., safety and security, food/ hydration/ shelter, health and medical, transportation, water systems). When multiple systems that are fundamental to community functioning are simultaneously disrupted, the cumulative effect becomes the systematic undermining of civilian life-support systems. This pattern reveals a strategic logic underlying military actions to be the disruption of community lifelines to create conditions where civilian populations face compound survival challenges that extend well beyond immediate physical damage. Whether intentional or not, the practical effect of disrupting infrastructure that aligns with community lifelines is to render civilian areas increasingly uninhabitable, fundamentally challenging the principles of civilian protection enshrined in international humanitarian law. The significance of this pattern extends beyond immediate humanitarian concerns to fundamental questions about the future of civilian protection in armed conflict and the adequacy of current international legal frameworks to address systematic infrastructure destruction as a method of warfare.

### Understanding systems producing patient outcomes

Our findings also emphasize the necessity of contextualizing patient outcomes within the systems that produce them. In Gaza, the very mechanisms responsible for generating the humanitarian crisis also prevent efforts to mitigate its effects (Fig 2) by impeding the delivery of humanitarian aid [38] and obstructing medical evacuations [39]. Such realities reshape the healthcare journey and transform it into one fraught with delays, risks, and barriers, significantly altering the usual trajectory of disease progression. For example, when patients who are critically ill because of residential destruction and displacement into crowded camps without access to clean water are also prevented from being transported to receive higher levels of care, it leads to avoidable multi-organ failure and death (Fig 2). Understanding patient trajectories within systems that create, shape, and sustain them is essential to designing interventions that meet immediate clinical needs, address the underlying drivers of those needs, and overcome barriers to care and recovery [40].

### Implications for humanitarian action

This study offers potentially significant implications for how humanitarian organizations conceptualize and respond to crises in conflict zones. The AoS map calls into question traditional sector-based humanitarian responses, which compartmentalize interventions into discrete clusters and fundamentally misalign with the lived experiences of affected populations who encounter these disruptions as a system of interconnected survival challenges.

The identification of many inter-related pathways linking conflict-related disruptions to health outcomes underscores the necessity for integrated, multi-sectoral humanitarian programming. Rather than addressing food insecurity, water and sanitation, protection, education, shelter, nutrition, and health as separate clusters, humanitarian aid might benefit from cross-sectoral responses that recognize and address the cascading relationships between these factors. For instance, our findings show that women’s inability to access self-care due to caregiving burdens directly impacts their capacity to provide care for children, creating downstream effects on pediatric health outcomes. This suggests that psychosocial support and respite care for women could have multiplier effects across health outcomes.

Additionally, the centrality of women’s health with 97% of pathways intersecting with women-related nodes demands a fundamental reorientation of humanitarian priorities. Current humanitarian frameworks often treat women’s health as a specialized sector rather than recognizing it as a critical mediator of population health outcomes. Our findings suggest that investing in women’s physical and mental health, reducing their caregiving burdens, and enabling their participation in social roles could serve as leverage points for improving overall community health outcomes.

To facilitate inter-sectoral collaboration, constructing an AoS map as part of ongoing needs assessment using the FAIR Framework could offer a valuable tool for humanitarian assessment and program design. By mapping the factors shaping health outcomes from the perspective of affected communities, humanitarian organizations can identify and monitor critical nodes and pathways where interventions might have the greatest cascading positive effects. This participatory approach also reduces the epistemic violence inherent in externally-imposed humanitarian frameworks by centering community knowledge and experiences.

### Implications for Human Rights and International Humanitarian Law

The systematic nature of disruptions documented in this study raise significant concerns under international humanitarian law (IHL). In the affected community’s experience, military actions lead to direct mortality and morbidity while also creating a cascade of indirect effects that transform preventable diseases into fatal outcomes.

Under the Fourth Geneva Convention and Additional Protocol I, parties to conflict have specific obligations to ensure the survival of civilian populations in occupied territories and in conflict zones. Article 55 of Additional Protocol I requires occupying powers to ensure “food and medical supplies of the population” [41]. Our findings reveal how the prevention of food transport forced populations to survive by consuming toxic materials, such as animal feed directly contributing to hepatotoxicity and severe acute malnutrition, suggesting potential violations of these provisions.

The targeting of water infrastructure noted by community members in our study and confirmed by other studies [31,34–36], which included bombing and destruction of water treatment plants, aquifers, wells, and desalination plants, and municipal water storage tanks contravene Article 54(2) of Additional Protocol I, which prohibits attacks on “objects indispensable to the survival of the civilian population” [41]. The resulting water scarcity created conditions for waterborne disease transmission, directly contributing to the Hepatitis A cases that became fatal due to concurrent healthcare system destruction.

Article 18 of the Fourth Geneva Convention requires parties to ensure that “civilian hospitals...may in no circumstances be the object of attack” [42]. The bombing and destruction of healthcare facilities, as noted in our study, combined with blockade of medical supplies and prevention of healthcare workers to travel, reveals a pattern of actions that systematically undermined the healthcare system’s capacity to manage preventable diseases. The denial of medical evacuations, preventing patients from accessing life-saving care, constitutes a violation of Article 17 of the Fourth Geneva Convention, which requires parties to “endeavour to conclude local agreements for the removal from besieged or encircled areas of wounded, sick, infirm, and aged persons, children and maternity cases” [42].

Relevant to special protections under IHL, our analysis reveals gendered impacts, with women bearing disproportionate burdens that compromise their health and ability to provide care. Article 76 of Additional Protocol I requires that “women shall be the object of special respect and shall be protected in particular against rape, forced prostitution and any other form of indecent assault” [41]. While our study did not attempt to document sexual violence, it reveals how conflict-induced conditions systematically undermine women’s health and dignity through forced survival choices and overwhelming caregiving burdens.

The transformation of a preventable disease into a fatal outcome through the systematic destruction of civilian infrastructure and denial of basic necessities suggests a pattern that may amount to collective punishment, prohibited under Article 33 of the Fourth Geneva Convention [42]. Moreover, the scale and systematic nature of these actions, as revealed through the 19 source nodes of military actions against civilian populations and infrastructure, raise questions about potential war crimes under the Rome Statute of the International Criminal Court, particularly Article 8(2)(b)(xxv) regarding “intentionally using starvation of civilians as a method of warfare by depriving them of objects indispensable to their survival, including willfully impeding relief supplies as provided for under the Geneva Conventions” [43].

These findings underscore the urgent need for independent investigation and accountability mechanisms to assess whether the documented patterns constitute violations of IHL. The evidence provided by the FAIR Framework offers a novel approach to documenting systematic impacts of military actions on civilian populations, potentially supporting legal efforts to ensure compliance with international humanitarian law and protect civilian populations in conflict zones.

### Theorizing the findings

When the findings of this study are examined through a theoretical lens, we find that disruptions of seemingly neutral services, like healthcare, water, and food systems are neither random nor incidental; rather, they reflect deliberate strategies of control, exertion of power and collective punishment.

Theoretical frameworks that explain these findings include those related to structural violence and population control. The destruction of a population’s means of survival, combined with the deprivation of necessary resources to respond to that destruction, creates engineered contexts of systematic deprivation, in which populations are compelled into survival strategies that ultimately become pathways to death. This constitutes what Mbembe terms “necropolitical” governance: the creation of conditions where survival strategies themselves become pathogenic [44]. The study participants’ experiences of being forced to choose between starvation and consuming hazardous substances exemplifies how conflict can engineer “death-worlds” as “forms of social existence in which vast populations are subjected to conditions of life conferring upon them the status of living dead” [44].

In a necropolitics framework the AoS map is a methodology to reveal conditions that determine who may live and who is pushed to death. The initiating causes of military activities targeting civilian infrastructure are “technologies of destruction” used to exercise sovereignty through the systematic administration of an entire populations’ life chances. The documentation of pathways connecting military actions to adverse health outcomes demonstrates necropower’s operation through “indirect massacre,” where preventable diseases become fatal through the calculated withdrawal of life-sustaining conditions. These “technologies of slow death” are the systematic erosion of life chances over time rather than immediate killing. By targeting not just healthcare facilities but the entire system that makes medical care possible, from power generation to medical supply chains to evacuation routes, necropower operates through “systemic violence” that renders entire populations vulnerable to death. It operates through colonial logics that determine which populations can be subjected to conditions that would be unthinkable if applied to others, effectively placing entire populations outside the bounds of political protection while justifying systematic destruction of life-sustaining systems as military necessity [44]. The applicability of a necropolitical lens to explain the current devastation of Gaza has previously been noted by Hanbali and others [45].

Additionally, the systematic destruction of conditions that make community life possible (i.e., agricultural systems, water infrastructure, educational institutions) suggests what Patrick Wolfe theorizes as “elimination” rather than mere displacement [46]. The systematic and thorough destruction of civilian community spaces (e.g., residences, hospitals, schools and universities, churches and mosques) suggests that displacement may not be the goal. Rather, within Wolfe’s theorizing, the goal appears to be making return impossible.

Situating our findings within social theories on population control and structural violence allows us to move beyond a humanitarian logic exclusively focused on emergency response. Rather, it reveals that structural violence is not merely an incidental backdrop to suffering, but a mechanism actively and intentionally instrumentalized to reshape the very conditions of life and death for populations under siege.

### Limitations

This study’s findings are shaped by several limitations inherent to research conducted in active conflict zones. The reliance on a modified FAIR Framework, while context-sensitive, required adaptations that may have influenced data comprehensiveness and participant engagement. For example, condensing multiple sessions into single encounters and substituting patient charts for vignettes might have constrained the depth of narrative enrichment and thematic analysis. Additionally, the destruction of records and infrastructure posed challenges to obtaining consistent data, potentially leading to gaps in the representation of community experiences. The persistent threat to participant safety, including risks associated with documentation, may have further limited the candor and scope of shared insights. The impact of these limitations on the study is that there may be additional nodes, edges, and pathways missing from the AoS map that would otherwise have contributed to a more complete representation of community experience.

Future research should address these challenges by exploring alternative ways to adapt participatory methodologies to the context of active war and expanding the scope to include broader geographic and temporal contexts. Additionally, as systems are often dynamic, future research can move beyond a cross-sectional snapshot of a system and explore how to represent changing systems as experienced by the affected communities. Representing a dynamic system over time can enable monitoring, analysis, development of projections, and facilitate a humanitarian response that is sensitive to and anticipatory of evolving needs on the ground.

## Conclusion

This study reveals that military action on civilian populations engineers systems of worsening morbidity and mortality. The FAIR Framework used in this study offers a participatory assessment tool that centers community knowledge and addresses epistemic violence inherent in externally imposed humanitarian frameworks. It also offers a novel evidentiary approach for documenting human rights violations and can guide development of interventions to protect civilian populations in conflict zones.

The study’s identification of the system of interconnected pathways linking conflict disruptions to health outcomes calls for a re-evaluation of traditional sector-based humanitarian responses. Specifically, it points to a much-needed paradigm shift toward integrated, multi-sectoral programming that recognizes women’s health as a critical leverage point for population-wide outcomes.

Documentation through the AoS map canonizes community experiences of potential violations across multiple provisions of international humanitarian law. It reveals how military actions engineer cascading pathways that transform preventable diseases into fatal outcomes. These findings underscore an urgent need for independent investigation and mechanisms for accountability. Most importantly, the study concludes that meaningful health improvements ultimately require addressing causal factors of ongoing military actions against civilian populations and infrastructure, rather than merely mitigating their downstream effects.

The theorizing of this study’s findings suggests the need for new conceptual frameworks that can capture how conflicts create conditions where the very act of survival becomes a pathway to morbidity and mortality. Future research should examine whether similar patterns emerge in other conflict settings and develop theoretical frameworks that can better account for this form of systematic disruption of civilian life-support systems.

Finally, in aggregate, these findings call for IHL to be more explicit that elimination of conditions suitable for life of a population is an indication of intent to eliminate that population. These findings further suggest that just as actions, regardless of intent, destroy civilian populations, IHL should be re-examined and revised to weigh actions independently consequential regardless of ability to establish intent.

## Supporting information

S1 File Participatory interviewing scripts and results. (S1_File.PDF)

S2 File List of pathways. (S2_File.CSV)
