# Supplementary material for: Architecture of systems affecting disease trajectories in a conflict zone: A community-centered systems inquiry in North Gaza
Source: PLOS Glob Public Health. 2025 Sep 10;5(9):e0004450. doi: 10.1371/journal.pgph.0004450 (PMC12422423; doi:10.1371/journal.pgph.0004450)
Supplement: S1 File — (S1_File.PDF) [file pgph.0004450.s001.pdf]

S1 File. Participatory modeling scripts and results

SCRIPTS:
CLINICAL NARRATIVE ELICITING & DEVELOPMENT SCRIPT - completed as part of the patient intake process and present in the medical chart:
Context: At the beginning of narrative collection or vignette development process
Purpose: To provide a space for care-givers and providers to narrate patient journey, with a focus on the biomedical aspects of the journey.
Time required during session: 2-3 hours
Materials needed: laptop, text-editor or other note-taking software
Inputs: Trajectory of patient condition as recounted by care-giver; provider notes from patient medical chart
Outputs: Narrative of patient trajectory; initial visualization of patient journey
Roles: Facilitator to introduce session and process; care-givers and providers to voice patient journey and health seeking experience
Steps:
1. INTRODUCING THE PROJECT: The facilitator introduces the purpose of the activity and the process of collecting the patient journey.
2. INTRODUCING NARRATIVE COLLECTION:
Open-ended Questions For Caregivers:
- Please tell us about your child's illness.
- Tell us how you knew when they were sick. What were the symptoms that you noticed, and when did you bring them to the clinic or hospital?
- Can you describe your child's health before they became ill?
- Walk us through the events from when your child first showed signs of illness to when they received medical care.
- How did your child's condition change over time?
- What treatments did your child receive, and how did they respond?
- Were there any complications that arose during your child's illness?
Open-ended Questions For Healthcare Providers:
- Can you describe your experience treating this child with Hepatitis A?
- What symptoms and signs did the patient present with upon arrival?
- How did the patient's condition progress during their time under your care?
- What medical interventions were provided, and how did the patient respond?
- Were there any challenges in managing their condition from a clinical perspective?

NARRATIVE ENRICHMENT & INITIAL ARCHITECTURE OF SYSTEMS CONSTRUCTION SCRIPT:
Context: After participants have finished recounting the patient journey.
Purpose: To branch out from the biomedical aspects of the journey and enrich the patient narrative by clarifying details and factors that shaped the patient's and caregiver's choices and trajectories.
Materials Needed: Laptop, text editor or other note-taking software.
Inputs: Medical chart of the patient journey with additional narratives from the patient's family and healthcare providers.

Outputs: Patient journey visualization annotated with socio-political context.
Roles:
- Facilitator: Introduces the session and guides the process.
- Participants: Caregivers, community members, and key informants who provide lived experiences to clarify causality and proximal causes of choices and factors in the patient's trajectory.

Steps

1. Preparation
- Review Materials:
  - Familiarize yourself with the initial patient narratives and medical charts.
  - Prepare any previous notes or visualizations created during earlier sessions.
- Set Up Workspace:
  - Ensure a comfortable environment conducive to open discussion.
  - Have your laptop and note-taking software ready for real-time documentation.

2. Introduction
- Explain the Purpose:
  - Welcome participants and thank them for their continued involvement.
  - Briefly recap the previous session and outline the goals for this session.
  - Emphasize the importance of understanding the socio-political context to enrich the patient journey narrative.
- Establish Ground Rules:
  - Reinforce confidentiality and respect.
  - Encourage honest and open sharing.
  - Remind participants that they can take breaks or pause the discussion if needed.

3. Eliciting Socio-Political Context
- Pose Open-Ended Questions to Caregivers:
  - "How did the situation in your community affect your ability to care for your child?"
  - "Were there any challenges you faced due to the conflict when seeking medical help?"
  - "Can you describe how the war impacted your daily life during your child's illness?"
  - "Did you experience any difficulties accessing resources like clean water, food, or medicine for your child?"
  - "How did transportation or safety concerns affect your ability to reach healthcare facilities?"
  - "Were there any support systems or community resources available to you?"
- Pose Open-Ended Questions to Healthcare Providers:
  - "In what ways did the conflict influence the healthcare system and your ability to provide care?"
  - "What barriers did you face in accessing medical supplies and equipment?"
  - "How did the destruction of infrastructure affect patient outcomes?"
  - "Can you describe any instances where socio-political factors directly impacted your patient's care?"
  - "What strategies did you employ to overcome these challenges?"
- Facilitate the Discussion:
  - Encourage participants to elaborate on their responses.
  - Use active listening and show empathy.
  - Allow the conversation to flow naturally, while gently steering it back to the patient journey when necessary.

4. Recording and Notetaking

- Document Responses:
  - Take detailed notes of participants' insights, focusing on specific factors and events they mention.
  - Capture direct quotes where impactful.
- Seek Clarification:
  - If a point is unclear, ask follow-up questions:
    - "Could you tell me more about that experience?"
    - "How did that situation make you feel?"
    - "What happened after that?"

5. Initial Coding and Deconstruction

- Deconstruct Narratives:
  - Break down the stories into discrete, inter-related elements:
    - Events (e.g., hospital visits, onset of symptoms)
    - Decisions made (e.g., seeking alternative treatments)
    - Socio-political factors (e.g., roadblocks, resource scarcity)

7. Developing the Initial Visualization

- Create a Conceptual Model:
  - Begin constructing a visual representation of the patient journey, incorporating both medical and clinical events and socio-political context along a chronological axis
  - Use a timeline or flowchart format.
- Map Out Factors:
  - Plot biomedical events along the timeline.
  - Annotate with socio-political factors at relevant points.
    - For example, indicate "Checkpoint delays" next to "Delay in reaching hospital."
- Illustrate Relationships:
  - Use arrows or connectors to show interactions between factors.
  - Highlight how socio-political influences affected decisions and outcomes.

8. Collaborative Refinement

- Present the Visualization:
  - Share the initial diagram with participants.
  - Explain how you've incorporated their insights.
- Invite Feedback:
  - Ask participants:
    - "Does this diagram accurately reflect your experience?"
    - "Are there any factors we've missed or misrepresented?"
    - "How can we improve this visualization to better tell your story?"
- Modify Accordingly:
  - Make real-time adjustments based on their input.
  - Ensure that the visualization resonates with their perspectives.

9. Finalization

- Review the Model Together:
  - Go through the updated diagram step by step.
  - Confirm that all participants agree with the final representation.
- Ensure Clarity and Accuracy:
  - Check that all factors are clearly labeled and relationships are well-defined.

- Simplify complex areas if needed for better understanding.
10. Documentation
- Save the Visualization:
  - Digitally capture the final diagram using scanning or photography.
  - If using digital tools, save the file securely.
- Record Additional Insights:
  - Note any overarching themes or significant points that emerged during the session.
  - Document participants' reflections on the process.
11. Closing the Session
- Express Gratitude:
  - Thank participants sincerely for their time and valuable contributions.
- Discuss Next Steps:
  - Briefly outline how the enriched narratives and visualizations will inform the study.
  - Explain any follow-up actions or future sessions.
- Provide Support Resources:
  - Offer information on support services if the discussion brought up any emotional distress.

NARRATIVE ANALYSIS & ITERATIVE DEVELOPMENT OF ARCHITECTURE OF SYSTEMS MODEL
• Context: After narrative / vignette is recorded and enriched, narrative analysis can begin
• Purpose: An in-depth exploration of patient journey annotated with socio-political context
• Time required during session: 2-3 hours
• Materials needed:
o Laptop
• Inputs: Patient journey visualization annotated with socio-political context
• Outputs:
Initial Coding Outputs:
Deconstructed Factors: Reviewing each factor in the diagram to examine if they can be further broken down into discrete inter-related elements.
  Code List: A comprehensive list of initial codes representing each identified factor.

Intermediate Coding Outputs:
- Categorization of Factors: Grouping related codes under broader categories or labels to create thematic clusters of causes (X-axis) and relationships (Y-axis).
- Relationships Between Categories: Identifying and mapping cause-effect relationships between categories.; Understanding how one category influences or leads to another.
- Conceptual Model Evolution: Reorganizing diagram and interconnections between categories; Using constant comparative analysis to refine and collapse categories where appropriate.
- Summary of Themes: Summarizing the various factors within broader themes to understand the overarching issues affecting patient journeys.

Advanced Coding Outputs:
- Synthesis of Common Concepts: Integrating findings across both patient narratives to identify common concepts and experiences; recognizing patterns and systemic issues that recur in different cases.

- Development of Super-Categories: Creating higher-level abstractions that encompass multiple categories
- Architecture of Systems Map: Improving patient journey map represent relationships between all identified factors and categories. This map illustrates how socio-political factors interact with biomedical aspects of the patient journey and reveals the conceptual model depicting the systemic influences on health outcomes.

Steps:
1. Preparation
   - Gather Materials:
     - Patient journey block diagram visualization on paper.
     - Transcribed narratives from caregivers and healthcare professionals.
     - Writing tools (markers, pens) for modifying the diagram.
   - Familiarize Yourself with the Narratives:
     - Read through the enriched narratives thoroughly to understand the patient's journey and the socio-political context.
   - Set Up Workspace:
     - Arrange a comfortable and organized workspace where you can easily modify the diagram during the analysis.
2. Initial Coding
   - Deconstruct the Patient Journey:
     - All factors were in the diagram were reviewed to evaluate if they should be further deconstructed into discrete, inter-related elements.
   - Modify the Diagram:
     - Any additional identified factors and relationships were added to the to the patient journey block diagram.
     - Different symbols were used to represent different types of factors (e.g., biomedical events, socio-political influences).
   - Create an Initial Code List:
     - Document all the factors added to the diagram as initial codes.
     - Ensure each code clearly represents a specific element from the narratives.
     - Through participant feedback ensure no additional code needs to be added to effectively represent patient journeys and lived experiences.
3. Intermediate Coding
   - Categorize Factors:
     - Group related codes on the diagram into broader categories or thematic clusters.
     - Label these categories directly on the diagram (e.g., "Healthcare Access Barriers," "Economic Challenges").
   - Identify Relationships Between Categories:
     - Draw arrows or lines to show cause-effect relationships between categories.
     - Illustrate how one category influences or leads to another on the diagram.
   - Refine the Conceptual Model:
     - Reorganize the diagram to reflect the interconnections between categories more clearly.
     - Use constant comparative analysis to refine and collapse overlapping categories.

- Summarize Themes:
  - Note overarching themes that emerge from the categories and relationships.
  - Add these summaries to the diagram where appropriate.

4. Advanced Coding
   - Synthesize Common Concepts:
     - Integrate findings across both patient narratives to identify common patterns and systemic issues.
     - Highlight these common concepts on the diagram.
   - Develop Super-Categories:
     - Create higher-level abstractions that encompass multiple categories (e.g., "Systemic Healthcare Barriers").
     - Label these super-categories on the diagram.
   - Finalize the Architecture of Systems Map:
     - Enhance the diagram to represent relationships between all identified factors, categories, and super-categories.
     - Ensure the diagram illustrates how socio-political factors interact with biomedical aspects of the patient journey.
     - Confirm that the conceptual model accurately depicts the systemic influences on health outcomes.
5. Finalization
   - Review the Diagram:
     - Examine the final diagram for accuracy and completeness.
     - Make any necessary adjustments to ensure clarity.
   - Document the Findings:
     - Take a clear photograph or scan of the finalized diagram.
     - Prepare accompanying notes or a brief report summarizing the key findings and themes.
   - Prepare for Next Steps:
     - Use the finalized Architecture of Systems Map to inform further analysis, reporting, or development of interventions.

COMMUNITY VALIDATED ARCHITECTURE OF SYSTEMS CONCEPTUAL MODEL SCRIPT:

• Context: After conducting narrative analysis, the facilitator will have summarized and visualized the relationships surfaced in the narrative analysis effort. This visualization represents the architecture of systems conceptual model. Now the architecture of systems conceptual model is examined with the participant to ensure that it accurately represents their narrative thoughts.
• Purpose: A validation of the summarization and visualization of systems and relationships as mentioned by participant in their narrative or vignette.
• Time required during session: 2-3 hours
• Materials needed: Laptop
• Inputs: Initial architectures of systems conceptual model (visualization)
• Outputs: Community validated architecture of systems conceptual model
• Roles: Facilitator to introduce session and process; Community member or key informants to voice lived experience
• Steps: The facilitator presents the architecture of systems conceptual model and explains that it is a visualization of relationships identified in the narratives from previous sessions. The facilitator goes

through each factor and relationship and asks the participant if the way it is represented accurately reflects their experience or how else the factors or relationships can be modified

Table A. List of Codes Comprising the Architecture of Systems Map
The following is a list of Codes Represented by the Broader Categories in the Community Validated Architecture of Systems Map.

	STARVATION	FORCED POPULATION MOVEMENTS; TRAMATIC INJURIES	EFFECT ON SANITATION & ENVIRONMENT	EFFECT ON WATER AVAILABILITY	EFFECT ON HEALTHCARE SYSTEM	EFFECT ON MATERNAL AND WOMEN'S HEALTH
**Precipitating factor: Bombing and destruction of people, infrastructure, and resources**		Bombing and destruction of camps and areas with displaced civilians<br><br>Bombing and destruction of homes, residential buildings, and civilian areas	Bombing and destruction of water treatment plants and sanitation infrastructure	Bombing and destruction of water treatment plants and sanitation infrastructure<br><br>Bombing and destruction of infrastructure used for water trucking<br><br>Bombing and destruction of aquifers, wells, and desalination plants<br><br>Bombing and destruction of municipal water storage tanks	Bombing and destruction of Markets and Supply Routes<br><br>Bombing and destruction of roads<br><br>Bombing and destruction of healthcare facilities	Blockade of aid; bureaucratic delays in humanitarian aid approval

**Precipitating factor: Deprivation of resources**	Preventing transport of food to population<br><br>Preventing transport of necessary nutrition sources			Blockade preventing entry of spare parts and tools<br><br>Shutdown of water lines	Blockade of transport of essential items (e.g., fuel)<br><br>Blockade of medical supplies<br><br>Blockade and Restrictions on patient travel and medical evacuation<br><br>Blockade and Prevention of healthcare workers to travel within and between Gaza regions	Lack of access to sanitary pads or tampons<br><br>Lack of access to contraceptives<br><br>Lack of prenatal care and nutrition

**Loss of resources necessary for survival**	Prolonged periods of unavailability of food<br><br>Lack of necessary nutrition sources<br><br>Loss of community gardens and local sources of food production	Homes and neighborhoods reduced to rubble; loss of homes and communities<br><br>Loss of stability; Repeated displacement	Destruction and loss of sewage pumps and treatment facilities	Contaminated water sources; Lack of clean water<br><br>Unable to treat contaminated Water Sources	Scarcity of essential goods<br><br>Destruction, loss, and collapse of healthcare services<br><br>Lack of fuel Shortage and depletion of critical lab / diagnostic supplies (e.g., reagents, urine test strips)<br><br>Lack of disposables: gloves (examination, surgical gloves, etc), gowns, gauze pads, sterilization supplies, syringes, urinary supplies, NG tubes, bandages and dressings, urine test strips, sutures<br><br>Shortage and depletion of medicines (e.g., anesthetics, antibiotics, analgesics, and other essential medicines)<br><br>Medical evacuation denied / attempts prevented Prevents physician with training in higher levels of care from coming to area of need to help	

**Sequelae of deprivation and loss of resources**	Starvation<br><br>Insufficient caloric and nutritional intake	Physical Dangers to Infants and Children<br><br>Injuries and Trauma<br><br>Population Displacement to Camps<br><br>Overcrowding<br><br>Increasing loss of critical items with each repeated displacement - loss of clothing, documents showing identity, home / land ownership, and other critical items brought along when fleeing bombings or attacks		Forces population to move to places where water is available<br><br>Population using contaminated water sources for drinking and hygiene<br><br>Destruction of laboratories preventing water quality testing<br><br>Inability to establish safe water and sanitation facilities<br><br>Significant reduction in water availability<br><br>Decline in crop irrigation and agricultural output<br><br>Collapse of livelihoods dependent on agriculture	Patients must risk lives to obtain necessities<br><br>Pt transport on foot, being carried, or by animal cart (e.g. donkey cart)<br><br>Increased distance to reach functional healthcare facilities<br><br>Threat to life along routes of travel to reach care<br><br>Not able to travel to higher level of care (e.g. across border)	Unplanned pregnancies<br><br>Use of makeshift materials (tent scraps, cloth) as pads
**Impact on population movement and actions: Forced population adaptations**		Fear of being prevented from returning home or fear of their land will be occupied and home annexed	Makeshift bathrooms without proper facilities	Inability to identify safe drinking water sources		Increased risk of infections (e.g., Toxic Shock Syndrome, rashes)

**Sequelae of forced population adaptations**		Some residents continue staying in areas of being bombed	Accumulation of human waste<br><br>Environmental contamination<br><br>Pollution of groundwater from sewage overflow	Inability to maintain hygiene in healthcare facilities	Prevents population mobility to seek care<br><br>Prevents timely transport and transfer of patients to higher level of care (e.g. ICU)	
**Risk factors for morbidity and mortality**		Facilitated transmission of communicable diseases	Increased risk of water-borne diseases and fecal-oral route diseases<br><br>Infection in individuals		Delayed presentation to health care environment<br><br>Sporadic ICU function<br><br>Manual ventilation of patients throughout the night<br><br>Increased infection risk<br><br>Inability to monitor patient status<br><br>Prevention of patient examination and intervention	Women presenting with severe symptoms (weakness, fever)

**Impact on care delivery**		Untreated or temporarily treated injuries (e.g. external fixation for fractures)			Delay in or inadequate provision of critically needed care<br><br>Delayed detection of complications<br><br>Inadequate management of patients<br><br>Inability to provide life-saving care<br><br>Need for referral to higher-level care facilities<br><br>Delayed or inadequate treatment	
**Disease trajectory**	Organ toxicity (e.g., hepatotoxicity)<br><br>Onset of starvation and acute malnutrition	Deaths from bombings and destruction of civilian structures<br><br>Traumatic injuries from bombings and destruction of civilian structures<br><br>Infections and complications	Preventable disease deaths<br><br>Progression to severe disease in vulnerable populations		Deterioration of Patient condition<br><br>Patients progressing to late stages of disease<br><br>Further decompensating patient requires even more urgent medical evacuation<br><br>Progression to multi-organ failure and death	Increased maternal health risks<br><br>Increased maternal and neonatal deaths<br><br>Birth complications (miscarriages, stillbirths, prematurity)<br><br>Caregivers unable to provide needed care effectively

The data generated and analyzed in this is included below in JSON format, with data to re-construct edges and nodes as provided in the article (e.g. reconstruction of the AoS map can be done by inputting the JSON object into GoJS software):

{ "class": "GraphLinksModel",
"nodeDataArray": [
{"key":1,"text":"Preventing transport of food to population","loc":"1518.8190877114214 -1466.1805024644582"},
{"key":2,"text":"Prolonged periods of unavailability of food","loc":"1519.008883371618 -974.8222470972615"},
{"key":3,"text":"Starvation","loc":"1567.9280932912527 -445.5004433998956"},
{"key":4,"text":"Consumption of toxic materials for food (e.g., animal feed)","loc":"1529.5562459506511 245.18764551702293"},
{"key":5,"text":"Organ toxicity (e.g., hepatotoxicity)","loc":"1529.0600307070367 2238.5613836218417"},
{"key":6,"text":"Onset of starvation and acute malnutrition","loc":"1739.0462884695892 2238.4116840827737"},
{"key":11,"text":"Preventing transport of necessary nutrition sources","loc":"1730.0090062014103 -1455.9420586995766"},
{"key":12,"text":"Lack of necessary nutrition sources","loc":"1730.0090062014103 -974.8222470972615"},
{"key":13,"text":"Insufficient caloric and nutritional intake","loc":"1802.5534497441356 -438.0318638477053"},
{"key":16,"text":"Blockade of transport of essential items (e.g., fuel)","loc":"5308.996941093129 -1477.734840142988"},
{"key":17,"text":"Lack of fuel","loc":"5212.88835810418 -1034.3365009903946"},
{"key":18,"text":"Sporadic ICU function","loc":"5213.8151634165215 1357.1955013350034"},
{"key":19,"text":"Manual ventilation of patients throughout the night","loc":"5213.936270694999 1435.4757258696188"},
{"key":23,"text":"Deterioration of Patient condition","loc":"5606.39851812288 2228.7117854191847"},
{"key":24,"text":"Bombing and destruction of homes, residential buildings, and civilian areas","loc":"2026.5721688911915 -1695.4469135013737"},
{"key":25,"text":"Population Displacement to Camps","loc":"2443.2947703039413 -468.5274643215511"},
{"key":26,"text":"Overcrowding","loc":"2439.3852113437893 -344.8763652938592"},
{"key":27,"text":"Facilitated transmission of communicable diseases","loc":"2461.4227479226793 1339.9739680806995"},
{"key":29,"text":"Prevents timely transport and transfer of patients to higher level of care (e.g. ICU)","loc":"5114.011874693816 777.7800411449665"},
{"key":32,"text":"Bombing and destruction of camps and areas with displaced civilians","loc":"2673.8539532505715 -1695.4469135013733"},
{"key":33,"text":"Loss of stability; Repeated displacement","loc":"2673.8539532505715 -974.8222470972615"},
{"key":34,"text":"Inability to establish safe water and sanitation facilities","loc":"3991.4619670159177 -481.40295372735983"},
{"key":35,"text":"Increased risk of water-borne diseases and fecal-oral route diseases","loc":"3030.48872659633 1335.1167974562081"},

{"key":37,"text":"Threat to life along routes of travel to reach care","loc":"5051.828829567069 -371.7507943198299"},
{"key":38,"text":"Bombing and destruction of healthcare facilities","loc":"5340.925864207696 -1703.5751468387762"},
{"key":39,"text":"Increased distance to reach functional healthcare facilities","loc":"5089.182318464779 -488.09923910049184"},
{"key":46,"text":"Blockade of medical supplies","loc":"5545.560116664124 -1476.4785556539287"},
{"key":47,"text":"Shortage and depletion of critical lab / diagnostic supplies (e.g., reagents, urine test strips)","loc":"5376.466587916565 -906.692790262828"},
{"key":48,"text":"Inability to monitor patient status","loc":"5603.338721147732 1366.7776877742974"},
{"key":49,"text":"Delayed detection of complications","loc":"5606.674405601126 1877.0544048606998"},
{"key":51,"text":"Shortage and depletion of medicines (e.g., anesthetics, antibiotics, analgesics, and other essential medicines)","loc":"5757.823153387356 -885.7963803980429"},
{"key":52,"text":"Inadequate management of patients","loc":"5944.3853175412105 1880.4562494421425"},
{"key":53,"text":"Patients progressing to late stages of disease","loc":"5943.99593264217 2228.5893553854276"},
{"key":55,"text":"Blockade and Restrictions on patient travel and medical evacuation","loc":"5808.263632038413 -1479.4574941802343"},
{"key":56,"text":"Need for referral to higher-level care facilities","loc":"6296.761208346462 1880.9471006060796"},
{"key":57,"text":"Further decompensating patient requires even more urgent medical evacuation","loc":"6148.63985110941 2219.7845002412087"},
{"key":58,"text":"Inability to provide life-saving care","loc":"6110.200286362687 1879.444712963166"},
{"key":59,"text":"Progression to multi-organ failure and death","loc":"6353.035987814044 2217.9893925446704"},
{"key":61,"text":"Lack of access to female hygiene supplies (e.g. sanitary pads, tampons)","loc":"6423.502064246742 -1007.400299591671"},
{"key":62,"text":"Use of makeshift materials (tent scraps, cloth) as pads","loc":"6366.653081790085 228.2397581004684"},
{"key":64,"text":"Increased risk of infections (e.g., Toxic Shock Syndrome, rashes)","loc":"6317.024178968754 1367.390875762883"},
{"key":65,"text":"Women presenting with severe symptoms (weakness, fever)","loc":"6559.0509381921665 1367.390875762883"},
{"key":66,"text":"Delayed or inadequate treatment","loc":"6491.796625087424 1889.5429057399517"},
{"key":83,"text":"Destruction, loss, and collapse of healthcare services","loc":"5013.1632114729355 -974.8222470972612"},
{"key":84,"text":"Lack of prenatal care and nutrition","loc":"6814.62616648779 -1000.5748870424619"},
{"key":85,"text":"Birth complications (miscarriages, stillbirths, prematurity)","loc":"6985.6229597202055 2177.7959096355917"},
{"key":86,"text":"Increased maternal and neonatal deaths","loc":"6786.069451180672 2182.4463714879785"},
{"key":87,"text":"Lack of access to contraceptives","loc":"6621.806935692975 -1001.1355099635783"},
{"key":88,"text":"Unplanned pregnancies","loc":"6512.343474532491 -479.5322111686643"},
{"key":89,"text":"Increased maternal health risks","loc":"6571.868781658506 2185.309850734274"},
{"key":121,"text":"Homes and neighborhoods reduced to rubble; loss of homes and communities","loc":"2439.777390543199 -950.4375470850541"},

{"key":122,"text":"Physical Dangers to Infants and Children","loc":"2244.089244688083 -466.961255078995"},
{"key":123,"text":"Injuries and Trauma","loc":"2182.6997749883267 1361.6318617729646"},
{"key":125,"text":"Infections and complications","loc":"2276.963783872456 2239.090002489739"},
{"key":126,"text":"Bombing and destruction of Markets and Supply Routes","loc":"4760.303319349806 -1703.5751468387766"},
{"key":127,"text":"Scarcity of essential goods","loc":"4530.42512223233 -974.8222470972611"},
{"key":128,"text":"Patients must risk lives to obtain necessities","loc":"4503.992139864281 210.3529274495055"},
{"key":129,"text":"Exposure to danger (bombings, snipers)","loc":"4503.992139864281 1289.1511345910737"},
{"key":130,"text":"Potential for injury or death while seeking necessities","loc":"4613.356567843577 2235.625592013711"},
{"key":-113,"text":"Prevents population mobility to seek care","loc":"4840.933962755867 779.5208609933467"},
{"key":-117,"text":"Increasing loss of critical items with each repeated displacement - loss of clothing, documents showing identity, home / land ownership, and other critical items brought along when fleeing bombings or attacks","loc":"2657.3272213090972 -400.5806435843615"},
{"key":-119,"text":"Delayed presentation to health care environment","loc":"4976.317385337136 1371.8409925714723"},
{"key":-120,"text":"Initial presentation to health care facility is at critical / decompensating / advanced stage of condition","loc":"4949.097553357047 2234.905647360726"},
{"key":-121,"text":"Delay in or inadequate provision of critically needed care","loc":"5164.149228626561 1870.163585463343"},
{"key":-109,"text":"Untreated or temporarily treated injuries (e.g. external fixation for fractures)","loc":"2283.634752823567 1905.3583990345535"},
{"key":-110,"text":"PRECIPITATING FACTOR: DEPRIVATION OF RESOURCES","loc":"1255.130030920766 -1449.9240357896533"},
{"key":-111,"text":"PRECIPITATING FACTOR: \nBOMBING & DESTRUCTION OF PEOPLE, INFRASTRUCTURE & RESOURCES","loc":"1266.0524399783833 -1675.1805698574558"},
{"key":-112,"text":"SEQUELAE OF DEPRIVATION AND LOSS OF RESOURCES","loc":"1270.6923401859028 -431.4091649643631"},
{"key":-115,"text":"IMPACT ON POPULATION MOVEMENT & ACTIONS: FORCED POPULATION ADAPTATIONS","loc":"1263.285011576376 241.44337883535377"},
{"key":-118,"text":"Loss of community gardens and local sources of food production","loc":"1920.1000022744572 -958.5657804224567"},
{"key":-122,"text":"LOSS OF RESOURCES NECESSARY FOR SURVIVAL","loc":"1269.4610170176381 -966.6940137598591"},
{"key":-116,"text":"Pt transport on foot, being carried, or by animal cart (e.g. donkey cart)","loc":"4845.179102024899 -489.1982965426814"},
{"key":-124,"text":"IMPACT ON CARE DELIVERY","loc":"1266.9497593774859 1889.1019323597488"},
{"key":-125,"text":"IMPACT ON DISEASE TRAJECTORY","loc":"1270.27857110667 2239.090002489739"},
{"key":-126,"text":"RISK FACTORS FOR MORBIDITY & MORTALITY","loc":"1265.1541598307706 1377.8883284477693"},
{"key":-114,"text":"Bombing and destruction of roads","loc":"5013.1632114729355 -1711.7033801761786"},
{"key":-123,"text":"Not able to travel to higher level of care (e.g. across border)","loc":"5932.3720994860405 -474.50940202177867"},

{"key":-127,"text":"Fear of being prevented from returning home or fear their land will be occupied and home annexed","loc":"2631.6811227340527 -175.9521181694177"},
{"key":-128,"text":"Contaminated water sources; Lack of clean water","loc":"3304.5998449347867 -966.6940137598591"},
{"key":-129,"text":"Forces population to move to places where water is available","loc":"3311.6077208185366 209.5045409780305"},
{"key":-131,"text":"Medical evacuation denied / attempts prevented","loc":"5947.48945483272 -999.2720662542685"},
{"key":-132,"text":"Blockade and Prevention of healthcare workers to travel within and between Gaza regions ","loc":"6137.586703944437 -1490.6804040786005"},
{"key":-133,"text":"Prevents physician with training in higher levels of care from coming to area of need to help","loc":"6137.586703944437 -902.0528470728475"},
{"key":-134,"text":"DESTRUCTION OF MATERNAL AND WOMEN'S HEALTH","loc":"6730.361966167866 2410.2403051048377"},
{"key":-136,"text":"Lack of disposables: gloves (examination, surgical gloves, etc), gowns, gauze pads, sterilization supplies, syringes, urinary supplies, NG tubes, bandages and dressings, urine test strips, sutures","loc":"5576.222462308365 -1063.0701393807412"},
{"key":-138,"text":"Increased infection risk","loc":"5413.695264596047 1364.0217949084933"},
{"key":-139,"text":"Prevention of patient examination and intervention","loc":"5781.258809370965 1367.390875762883"},
{"key":-135,"text":"Progression to severe disease in vulnerable populations","loc":"3119.786555064968 2219.468145969322"},
{"key":-137,"text":"Infection in individuals","loc":"3033.6347196470574 1438.6908535652333"},
{"key":-142,"text":"Unable to treat contaminated water sources","loc":"3489.8300908688593 -465.6065102835803"},
{"key":-143,"text":"Bombing and destruction of water treatment plants and sanitation infrastructure","loc":"3233.159749768654 -1695.4469135013742"},
{"key":-145,"text":"Environmental contamination","loc":"3038.9427093542004 766.1334254666173"},
{"key":-146,"text":"Accumulation of human waste","loc":"2854.52098131053 767.2049311067325"},
{"key":-148,"text":"Makeshift bathrooms without proper facilities","loc":"3029.6509034657115 239.67134650293315"},
{"key":-149,"text":"Destruction and loss of sewage pumps and treatment facilities","loc":"3062.4415948614273 -966.6940137598589"},
{"key":-150,"text":"Pollution of groundwater from sewage overflow","loc":"3198.274622966985 769.8571553626697"},
{"key":-153,"text":"Preventable disease deaths","loc":"2867.881427993577 2225.527389461281"},
{"key":-155,"text":"Blockade preventing entry of spare parts and tools","loc":"3705.5757590160088 -1459.5013873140092"},
{"key":-156,"text":"Destruction of laboratories preventing water quality testing","loc":"3738.559143998786 -966.6940137598591"},
{"key":-157,"text":"Significant reduction in water availability","loc":"4012.233603164614 -366.273697366383"},
{"key":-158,"text":"Shutdown of water lines","loc":"3918.4378367210034 -1464.4170650745164"},
{"key":-159,"text":"Bombing and destruction of infrastructure used for water trucking","loc":"3701.0340948924736 -1695.4469135013742"},
{"key":-160,"text":"Bombing and destruction of aquifers, wells, and desalination plants","loc":"4074.693672172137 -1695.4469135013737"},

{"key":-161,"text":"Bombing and destruction of municipal water storage tanks","loc":"4328.543097589042 -1695.4469135013746"},
{"key":-162,"text":"Decline in crop irrigation and agricultural output","loc":"4231.447399529948 -465.6065102835803"},
{"key":-163,"text":"Collapse of livelihoods dependent on agriculture","loc":"4231.447399529948 -273.96658629976906"},
{"key":-165,"text":"Inability to maintain hygiene in healthcare facilities","loc":"3744.990479448005 -358.1454640289806"},
{"key":-151,"text":"Population using contaminated water sources for drinking and hygiene","loc":"3489.8300908688593 255.35836767634385"},
{"key":-169,"text":"Inability to identify safe drinking water sources","loc":"3739.997994401618 -465.60651028358035"},
{"key":-152,"text":"Blockade of aid; bureaucratic delays in humanitarian aid approval","loc":"6443.711732998204 -1485.0995337682593"},
{"key":-141,"text":"DESTRUCION OF HEALTHCARE SYSTEM","loc":"5536.507632834016 2419.3401211137384"},
{"key":-144,"text":"FORCED POPULATION MOVEMENTS; TRAMATIC INJURIES; DEATHS","loc":"2395.0162048825855 2392.9406303030987"},
{"key":-166,"text":"DESTRUCTION OF WATER SYSTEMS","loc":"3702.399514317622 2418.082695594669"},
{"key":-154,"text":"MALNUTRITION & STARVATION","loc":"1604.3005878077813 2411.7971217263316"},
{"key":-140,"text":"DESTRUCTION OF SANITATION SYSTEMS & ENVIRONMENT","loc":"2871.456753903552 2411.797236817221"},
{"key":-167,"text":"INCREASING NEED","loc":"1590.2982610136032 -840.6443139476352"},
{"key":-170,"text":"DESTRUCTION & LOSS OF RESOURCES TO ADDRESS NEED","loc":"1776.5003505125705 -840.6443139476352"},
{"key":-164,"text":"[INCREASING NEED] X [DESTRUCTION & LOSS OF RESOURCES TO ADDRESS NEED]","loc":"1672.6151547061231 -734.0514161827932"},
{"key":-172,"text":"DESTRUCTION & LOSS OF RESOURCES TO ADDRESS NEED","loc":"2496.9190216923926 1792.8671795547025"},
{"key":-173,"text":"INCREASING NEED","loc":"2089.7530646767077 1789.7802894006031"},
{"key":-147,"text":"DESTRUCTION & LOSS OF RESOURCES TO ADDRESS NEED","loc":"3544.4927967613785 1201.0776521347434"},
{"key":-175,"text":"INCREASING NEED","loc":"3368.0352855961746 1201.1762423469438"},
{"key":-176,"text":"INCREASING NEED","loc":"6519.955598794647 764.5420956250329"},
{"key":-178,"text":"DESTRUCTION & LOSS OF RESOURCES TO ADDRESS NEED","loc":"6732.372145772143 756.3252136680309"},
{"key":-179,"text":"SEQUELAE OF FORCED POPULATION ADAPTATIONS","loc":"1267.8214561450864 772.9602115104793"},
{"key":-180,"text":"DESTRUCTION & LOSS OF RESOURCES TO ADDRESS NEED","loc":"3543.899428964149 -829.9374221777589"},
{"key":-181,"text":"INCREASING NEED","loc":"3116.2348978925215 -846.1938888525635"},
{"key":-182,"text":"Some residents continue staying in areas of being bombed","loc":"2628.4156274337597 375.7329603699028"},
{"key":-183,"text":"Deaths from bombings and destruction of civilian structures","loc":"1955.746266874531 2237.214409995302"},

{"key":-184,"text":"Traumatic injuries from bombings and destruction of civilian structures","loc":"2490.744306708567 2237.214409995302"},
{"key":-185,"text":"Caregivers unable to provide needed care effectively","loc":"6792.085579415774 2284.7430033866776"},
{"key":-186,"text":"INCREASING NEED","loc":"3056.7661550090897 1689.5581972457383"},
{"key":-187,"text":"DESTRUCTION & LOSS OF RESOURCES TO ADDRESS NEED","loc":"3238.0501002801125 1690.6416931271463"},
{"key":-189,"text":"[INCREASING NEED] X [DESTRUCTION & LOSS OF RESOURCES TO ADDRESS NEED]","loc":"2283.634752823567 1784.7389462173"},
{"key":-171,"text":"[INCREASING NEED] X [DESTRUCTION & LOSS OF RESOURCES TO ADDRESS NEED]","loc":"3133.927228685165 1800.9954128921047"},
{"key":-188,"text":"[INCREASING NEED] X [DESTRUCTION & LOSS OF RESOURCES TO ADDRESS NEED]","loc":"3449.4175506395914 1316.2557370706932"},
{"key":-174,"text":"[INCREASING NEED] X [DESTRUCTION & LOSS OF RESOURCES TO ADDRESS NEED]","loc":"3311.6077208185366 -821.8091888403563"},
{"key":-168,"text":"[INCREASING NEED] X [DESTRUCTION & LOSS OF RESOURCES TO ADDRESS NEED]","loc":"6626.0184936089 875.5178092730858"},
{"key":-177,"text":"Increased risk of communicable diseases for women","loc":"7120.592454815326 1367.390875762883"},
{"key":-190,"text":"Women foregoing self-care to be primary care giver for others","loc":"7308.331550668902 239.8767306661597"},
{"key":-191,"text":"Women with increased or primary responsibility for obtaining food, water, shelter, healthcare and other resources for self and children's survival","loc":"6901.390772080092 65.284420048176"},
{"key":-192,"text":"Worsening mental health for women","loc":"7360.666659607289 904.694988785769"},
{"key":-193,"text":"Women with unshared burden of responsibility for making decisions about displacement, finding aid and sustaining family","loc":"7107.692415142475 63.01161368140197"},
{"key":-195,"text":"Women with unshared burden of responsibility for family's survival and stability; and for being moral and cultural anchor for children","loc":"7527.431945975109 2.110477564432017"},
{"key":-196,"text":"Women with unshared burden of responsibility for navigating occupation forces to find status of children and spouses that were arrested or killed; providing stability and being moral and cultural anchor for children","loc":"7311.310821603961 -19.49654137315315"},
{"key":-194,"text":"Decreased women's participation in critical social roles","loc":"7554.808893346834 903.0103039112519"},
{"key":-197,"text":"Loss of healthcare workers","loc":"4797.710475711748 -974.8222470972609"},
{"key":-198,"text":"Women being surrogate parents: responsibility for survival and caregiving of children of relatives or neighbors lost to war","loc":"7709.42443865089 184.55041373023005"},
{"key":-199,"text":"Women with increased burden for fulfilling community support roles in camps or displaced settings","loc":"7871.506465853278 10.238710901834395"},
{"key":-200,"text":"Destruction of visions of a personal and social future for men and women, for students and academicians","loc":"7795.824611409811 -199.16920244315378"},
{"key":-201,"text":"Sense of hopelessness","loc":"7986.719550944455 953.0796887979759"},
{"key":-202,"text":"Bombing and destruction of universities and cessation of education","loc":"7794.261525788138 -1678.8521992089272"},
{"key":-204,"text":"Loss of electricity","loc":"5206.129413318878 -818.1759687127382"}
],
"linkDataArray": [

{"from":1,"to":2,"points":[1518.8190877114214,-1437.9240357896533,1518.8190877114214,-
1427.9240357896533,1518.8190877114214,-1220.50137478086,1519.008883371618,-
1220.50137478086,1519.008883371618,-1013.0787137720664,1519.008883371618,-
1003.0787137720664]},
{"from":3,"to":4,"points":[1567.9280932912527,-425.37221006249325,1567.9280932912527,-
415.37221006249325,1567.9280932912527,-108.28463227883867,1529.5562459506511,-
108.28463227883867,1529.5562459506511,198.80294550481585,1529.5562459506511,208.80294550
481585]},
{"from":4,"to":5,"points":[1529.5562459506511,281.5723455292299,1529.5562459506511,291.572345
5292299,1529.5562459506511,1245.9386312381334,1529.0600307070367,1245.9386312381334,1529
.0600307070367,2200.304916947037,1529.0600307070367,2210.304916947037]},
{"from":11,"to":12,"points":[1730.0090062014103,-1419.5573586873697,1730.0090062014103,-
1409.5573586873697,1730.0090062014103,-1211.318036229718,1730.0090062014103,-
1211.318036229718,1730.0090062014103,-1013.0787137720662,1730.0090062014103,-
1003.0787137720662]},
{"from":13,"to":6,"points":[1802.5534497441356,-409.7753971729006,1802.5534497441356,-
399.7753971729006,1802.5534497441356,900.189910117534,1739.0462884695892,900.18991011753
4,1739.0462884695892,2200.155217407969,1739.0462884695892,2210.155217407969]},
{"from":16,"to":17,"points":[5308.996941093129,-1441.3501401307813,5308.996941093129,-
1431.3501401307813,5308.996941093129,-1247.907437229289,5212.88835810418,-
1247.907437229289,5212.88835810418,-1064.4647343277968,5212.88835810418,-
1054.4647343277968]},
{"from":18,"to":19,"points":[5213.8151634165215,1377.3237346724056,5213.8151634165215,1387.32
37346724056,5213.8151634165215,1388.2073802649086,5213.936270695,1388.2073802649086,5213
.936270695,1389.0910258574118,5213.936270695,1399.0910258574118]},
{"from":25,"to":26,"points":[2412.7637390132513,-440.2709976467464,2412.7637390132513,-
422.2709976467464,2412.7637390132513,-398.63779813900396,2419.5138200474025,-
398.63779813900396,2419.5138200474025,-375.0045986312615,2419.5138200474025,-
365.0045986312615]},
{"from":26,"to":27,"points":[2409.578124399209,-324.74813195645686,2409.578124399209,-
298.74813195645686,2409.578124399209,497.4205680560178,2461.4227479226793,497.4205680560
178,2461.4227479226793,1293.5892680684924,2461.4227479226793,1303.5892680684924]},
{"from":33,"to":34,"points":[2734.4371187372253,-946.5657804224569,2734.4371187372253,-
936.5657804224569,2734.4371187372253,-936.3185488329851,2734.4371187372253,-
936.3185488329851,2734.4371187372253,-920.3185488329851,3855.3354844755554,-
920.3185488329851,3855.3354844755554,-481.4029537273599,3902.5164256340818,-
481.4029537273599,3912.5164256340818,-481.4029537273599]},
{"from":34,"to":35,"points":[3991.4619670159177,-445.0182537151528,3991.4619670159177,-
427.0182537151528,3991.4619670159177,-430.0829020928786,3991.4619670159177,-
430.0829020928786,3991.4619670159177,-422.0829020928786,3215.3354844755554,-
422.0829020928786,3215.3354844755554,289.9170979071214,3111.3354844755554,289.9170979071
214,3111.3354844755554,801.9170979071214,3058.416880588029,801.9170979071214,3058.416880
588029,1280.6038641065988,3058.416880588029,1290.6038641065988]},
{"from":38,"to":39,"points":[5366.662713674656,-1667.1904468265689,5366.662713674656,-
1649.1904468265689,5366.662713674656,-1648.318548832985,5151.335484475555,-
1648.318548832985,5151.335484475555,-928.318548832985,5089.182318464779,-
928.318548832985,5089.182318464779,-534.4839391126987,5089.182318464779,-
524.4839391126987]},

{"from":46,"to":47,"points":[5506.051243678284,-1448.2220889791238,5506.051243678284,-
1438.222088979124,5506.051243678284,-1203.7780229644823,5376.466587916565,-
1203.7780229644823,5376.466587916565,-969.3339569498409,5376.466587916565,-
959.3339569498407]},
{"from":47,"to":48,"points":[5376.466587916565,-854.0516235758163,5376.466587916565,-
844.0516235758163,5376.466587916565,242.23479876183825,5603.338721147732,242.23479876183
825,5603.338721147732,1328.5212210994928,5603.338721147732,1338.5212210994928]},
{"from":48,"to":49,"points":[5603.338721147732,1395.034154449102,5603.338721147732,1405.03415
4449102,5603.338721147732,1621.9160463174987,5606.674405601126,1621.9160463174987,5606.6
74405601126,1838.7979381858952,5606.674405601126,1848.7979381858952]},
{"from":46,"to":51,"points":[5585.068989649963,-1448.2220889791238,5585.068989649963,-
1438.222088979124,5585.068989649963,-1197.3939347007904,5757.823153387356,-
1197.3939347007904,5757.823153387356,-956.5657804224569,5757.823153387356,-
946.5657804224569]},
{"from":51,"to":52,"points":[5757.823153387356,-825.0269803736287,5757.823153387356,-
815.0269803736287,5757.823153387356,509.52228452815336,5944.3853175412105,509.5222845281
5336,5944.3853175412105,1834.0715494299354,5944.3853175412105,1844.0715494299354]},
{"from":52,"to":53,"points":[5944.3853175412105,1916.8409494543496,5944.3853175412105,1926.84
09494543496,5944.3853175412105,2058.586919082486,5943.99593264217,2058.586919082486,5943
.99593264217,2190.3328887106227,5943.99593264217,2200.3328887106227]},
{"from":61,"to":62,"points":[6423.502064246742,-962.8873662420616,6423.502064246742,-
952.8873662420616,6423.502064246742,-952.3185488329851,6366.653081790085,-
952.3185488329851,6366.653081790085,181.85505808826133,6366.653081790085,191.85505808826
133]},
{"from":64,"to":65,"points":[6399.535768019047,1367.3908757628833,6409.535768019047,1367.3908
757628833,6433.369517351212,1367.3908757628833,6433.369517351212,1367.390875762883,6457.
2032666833775,1367.390875762883,6467.2032666833775,1367.390875762883]},
{"from":65,"to":66,"points":[6559.0509381921665,1403.7755757750901,6559.0509381921665,1413.77
55757750901,6559.0509381921665,1632.5310074201186,6491.796625087424,1632.5310074201186,6
491.796625087424,1851.286439065147,6491.796625087424,1861.286439065147]},
{"from":83,"to":84,"points":[5098.448482163853,-974.8222470972611,5148.448482163853,-
974.8222470972611,5148.000076342598,-974.8222470972611,5148.000076342598,-
974.8222470972611,5476.000076342598,-974.8222470972611,5476.000076342598,-
958.8366204516709,5860.000076342598,-958.8366204516709,5860.000076342598,-
950.8366204516709,6036.000076342598,-950.8366204516709,6036.000076342598,-
958.8366204516709,6236.000076342598,-958.8366204516709,6236.000076342598,-
950.8366204516709,6700.000076342598,-950.8366204516709,6700.000076342598,-
1000.5748870424619,6722.767302657224,-1000.5748870424619,6732.767302657224,-
1000.5748870424619]},
{"from":84,"to":85,"points":[6814.62616648779,-972.3184203676573,6814.62616648779,-
962.3184203676573,6814.62616648779,-962.3184203676573,6804,-
962.3184203676573,6804,148,6967.853433047842,148,6967.853433047842,2123.411209623385,6967.
853433047842,2141.411209623385]},
{"from":83,"to":87,"points":[5098.448482163853,-980.8863637659623,5140.448482163853,-
980.8863637659623,5140.000076342598,-980.8863637659623,5140.000076342598,-
980.8863637659623,5476.000076342598,-980.8863637659623,5476.000076342598,-
958.8366204516709,5860.000076342598,-958.8366204516709,5860.000076342598,-
950.8366204516709,6036.000076342598,-950.8366204516709,6036.000076342598,-

958.8366204516709,6236.000076342598,-958.8366204516709,6236.000076342598,-
950.8366204516709,6516.000076342598,-950.8366204516709,6516.000076342598,-
991.7166877386434,6542.15881099815,-991.7166877386434,6552.15881099815,-
991.7166877386434]},
{"from":87,"to":88,"points":[6621.806935692975,-972.8790432887737,6621.806935692975,-
962.8790432887737,6621.806935692975,-736.2697438974201,6512.34347453249,-
736.2697438974201,6512.34347453249,-509.66044450606665,6512.34347453249,-
499.66044450606665]},
{"from":121,"to":122,"points":[2439.777390543199,-897.7963803980427,2439.777390543199,-
887.7963803980427,2439.777390543199,-696.5070510759213,2244.089244688083,-
696.5070510759213,2244.089244688083,-505.2177217537997,2244.089244688083,-
495.2177217537997]},
{"from":122,"to":123,"points":[2244.089244688083,-438.7047884041903,2244.089244688083,-
428.7047884041903,2244.089244688083,451.3994200156859,2182.6997749883267,451.39942001568
59,2182.6997749883267,1331.5036284355622,2182.6997749883267,1341.5036284355622]},
{"from":126,"to":127,"points":[4731.005193129103,-1667.1904468265689,4731.005193129103,-
1657.1904468265689,4731.005193129103,-1335.1345802993176,4530.42512223233,-
1335.1345802993176,4530.42512223233,-1013.0787137720661,4530.42512223233,-
1003.0787137720661]},
{"from":127,"to":128,"points":[4503.870391499095,-946.5657804224568,4503.870391499095,-
928.5657804224568,4503.870391499095,-378.23465982387796,4503.992139864281,-
378.23465982387796,4503.992139864281,172.09646077470086,4503.992139864281,182.0964607747
0086]},
{"from":128,"to":129,"points":[4503.992139864281,238.60939412431026,4503.992139864281,248.609
39412431026,4503.992139864281,749.7520310202897,4503.992139864281,749.7520310202897,4503
.992139864281,1250.894667916269,4503.992139864281,1260.894667916269]},
{"from":129,"to":130,"points":[4503.992139864281,1317.4076012658784,4503.992139864281,1327.40
76012658784,4503.992139864281,1758.3242466336912,4613.356567843577,1758.3242466336912,46
13.356567843577,2189.240892001504,4613.356567843577,2199.240892001504]},
{"from":37,"to":-113,"points":[5026.884722511405,-335.3660943076228,5026.884722511405,-
325.3660943076228,5026.884722511405,207.94915000545961,4840.933962755866,207.94915000545
961,4840.933962755866,741.264394318542,4840.933962755866,751.264394318542]},
{"from":-113,"to":-
119,"points":[4840.933962755867,807.7773276681514,4840.933962755867,817.7773276681514,4840.
933962755867,1071.6168101137082,4976.317385337137,1071.6168101137082,4976.317385337137,1
325.4562925592652,4976.317385337137,1335.4562925592652]},
{"from":-119,"to":-
120,"points":[4948.5931320005475,1408.2256925836793,4948.5931320005475,1426.2256925836793,
4948.5931320005475,1795.1809699599958,4949.097553357047,1795.1809699599958,4949.09755335
7047,2164.136247336312,4949.097553357047,2174.136247336312]},
{"from":39,"to":29,"points":[5117.034359073503,-451.7145390882848,5117.034359073503,-
433.7145390882848,5117.034359073503,-430.8366204516709,5156.832248228971,-
430.8366204516709,5156.832248228971,707.2671077953571,5156.832248228971,733.267107795357
1]},
{"from":37,"to":29,"points":[5076.772936622733,-335.3660943076228,5076.772936622733,-
325.3660943076228,5076.772936622733,194.95050674386715,5114.011874693815,194.95050674386
715,5114.011874693815,715.2671077953571,5114.011874693815,733.2671077953571]},

{"from":29,"to":-
121,"points":[5114.011874693816,822.2929744945759,5114.011874693816,832.2929744945759,5114.011874693816,836,5114.011874693816,836,5114.011874693816,1484,5135.415146086685,1484,5135.415146086685,1823.778885451136,5135.415146086685,1833.778885451136]},
{"from":58,"to":57,"points":[6110.200286362687,1907.7011796379707,6110.200286362687,1917.7011796379707,6110.200286362687,2037.4222565960838,6121.127432998245,2037.4222565960838,6121.127432998245,2157.143333554197,6121.127432998245,2167.143333554197]},
{"from":57,"to":59,"points":[6231.177105442906,2219.7845002412087,6241.177105442906,2219.7845002412087,6248.099069821835,2219.7845002412087,6248.099069821835,2217.9893925446704,6255.021034200763,2217.9893925446704,6265.021034200763,2217.9893925446704]},
{"from":57,"to":56,"points":[6176.152269220575,2167.143333554197,6176.152269220575,2157.143333554197,6176.152269220575,2042.2375670862418,6296.761208346462,2042.2375670862418,6296.761208346462,1927.3318006182867,6296.761208346462,1917.3318006182867]},
{"from":-
109,"to":125,"points":[2283.634752823567,1949.871332384163,2283.634752823567,1959.871332384163,2283.634752823567,2076.3524340995486,2257.105588885477,2076.3524340995486,2257.105588885477,2192.8335358149343,2257.105588885477,2210.8335358149343]},
{"from":24,"to":-118,"points":[1965.7536002527986,-1650.9339801517642,1965.7536002527986,-
1640.9339801517642,1965.7536002527986,-1327.0063469619151,1920.1000022744572,-
1327.0063469619151,1920.1000022744572,-1013.0787137720661,1920.1000022744572,-
1003.0787137720661]},
{"from":-118,"to":2,"points":[1844.555263504926,-943.7281359725871,1826.555263504926,-
943.7281359725871,1823.3354844755554,-943.7281359725871,1823.3354844755554,-
936.3185488329851,1647.3354844755554,-936.3185488329851,1647.3354844755554,-
974.8222470972615,1611.0443829443718,-974.8222470972615,1601.0443829443718,-
974.8222470972615]},
{"from":33,"to":-117,"points":[2643.562370507245,-946.5657804224569,2643.562370507245,-
912.5657804224569,2643.562370507245,-712.2784953591221,2657.3272213090977,-
712.2784953591221,2657.3272213090977,-511.99121029578737,2657.3272213090977,-
501.99121029578737]},
{"from":-118,"to":12,"points":[1844.555263504926,-973.4034248723266,1834.555263504926,-
973.4034248723266,1823.1377142293888,-973.4034248723266,1823.1377142293888,-
974.8222470972615,1811.7201649538517,-974.8222470972615,1801.7201649538517,-
974.8222470972615]},
{"from":24,"to":25,"points":[2038.7358826188702,-1650.9339801517642,2038.7358826188702,-
1624.9339801517642,2038.7358826188702,-1628,2038.7358826188702,-1628,2038.7358826188702,-
884,2412.7637390132513,-884,2412.7637390132513,-506.7839309963558,2412.7637390132513,-
496.7839309963558]},
{"from":24,"to":121,"points":[2087.3907375295844,-1650.9339801517642,2087.3907375295844,-
1640.9339801517642,2087.3907375295844,-1327.0063469619151,2439.777390543199,-
1327.0063469619151,2439.777390543199,-1013.0787137720661,2439.777390543199,-
1003.0787137720661]},
{"from":32,"to":33,"points":[2673.8539532505715,-1650.9339801517644,2673.8539532505715,-
1640.9339801517644,2673.8539532505715,-1327.0063469619154,2673.8539532505715,-
1327.0063469619154,2673.8539532505715,-1013.0787137720661,2673.8539532505715,-
1003.0787137720661]},
{"from":-
121,"to":23,"points":[5164.149228626561,1906.54828547555,5164.149228626561,1916.54828547555,

5164.149228626561,2053.501802109965,5551.8824674026655,2053.501802109965,5551.8824674026655,2190.45531874438,5551.8824674026655,2200.45531874438]},
{"from":19,"to":-121,"points":[5213.936270694999,1471.860425881826,5213.936270694999,1481.860425881826,5213.936270694999,1652.819655666481,5192.883311166437,1652.819655666481,5192.883311166437,1823.778885451136,5192.883311166437,1833.778885451136]},
{"from":49,"to":23,"points":[5606.674405601126,1905.3108715355045,5606.674405601126,1915.3108715355045,5606.674405601126,2044.8830951399423,5624.570535029618,2044.8830951399423,5624.570535029618,2174.45531874438,5624.570535029618,2200.45531874438]},
{"from":53,"to":23,"points":[5854.54216677303,2228.5893553854276,5844.54216677303,2228.5893553854276,5775.9003847148,2228.5893553854276,5775.9003847148,2228.7117854191847,5707.2586026565705,2228.7117854191847,5697.2586026565705,2228.7117854191847]},
{"from":39,"to":37,"points":[5061.330277856056,-451.7145390882848,5061.330277856056,-441.7145390882848,5061.330277856056,-429.9250167101609,5051.82882956707,-429.9250167101609,5051.82882956707,-418.1354943320369,5051.82882956707,-408.1354943320369]},
{"from":-114,"to":-116,"points":[5013.1632114729355,-1683.4469135013737,5013.1632114729355,-1673.4469135013737,5013.1632114729355,-1672.318548832985,4895.335484475555,-1672.318548832985,4895.335484475555,-936.318548832985,4845.179102024899,-936.318548832985,4845.179102024899,-543.7112298922907,4845.179102024899,-533.7112298922907]},
{"from":-116,"to":29,"points":[4845.179102024899,-444.6853631930721,4845.179102024899,-434.6853631930721,4845.179102024899,144.29087230114249,5071.191501158659,144.29087230114249,5071.191501158659,723.2671077953571,5071.191501158659,733.2671077953571]},
{"from":55,"to":-123,"points":[5808.263632038413,-1434.9445608306257,5808.263632038413,-1424.9445608306257,5808.263632038413,-1422.836620451671,5860.000076342598,-1422.836620451671,5860.000076342598,-950.836620451671,5932.3720994860405,-950.836620451671,5932.3720994860405,-520.8941020339857,5932.3720994860405,-510.8941020339858]},
{"from":-117,"to":-123,"points":[2747.4238933672027,-400.5806435843615,2757.4238933672027,-400.5806435843615,2759.3354844755554,-400.5806435843615,2759.3354844755554,-422.0829020928786,5183.335484475556,-422.0829020928786,5183.335484475556,-474.50940202177867,5830.794050779987,-474.50940202177867,5840.794050779987,-474.50940202177867]},
{"from":24,"to":-127,"points":[2063.0633100742266,-1650.9339801517642,2063.0633100742266,-1632.9339801517642,2063.0633100742266,-1632.318548832985,2063.0633100742266,-1632.318548832985,2063.0633100742266,-288.318548832985,2613.322998954756,-288.318548832985,2613.322998954756,-246.5932848564293,2613.322998954756,-228.5932848564293]},
{"from":33,"to":-127,"points":[2673.8539532505715,-946.5657804224569,2673.8539532505715,-920.5657804224569,2673.8539532505715,-920.3185488329851,2759.3354844755554,-920.3185488329851,2759.3354844755554,-288.3185488329852,2650.03924651335,-288.3185488329852,2650.03924651335,-246.59328485642948,2650.03924651335,-228.59328485642948]},
{"from":-117,"to":-127,"points":[2657.3272213090972,-299.17007687293574,2657.3272213090972,-289.17007687293574,2657.3272213090972,-275.88168086468255,2686.7554940719433,-275.88168086468255,2686.7554940719433,-262.5932848564294,2686.7554940719433,-228.59328485642942]},

{"from":-129,"to":-
127,"points":[3283.27544288478,173.1198409658234,3283.27544288478,163.1198409658234,3283.27
544288478,-175.9521181694177,3008.373592257659,-175.9521181694177,2733.471741630537,-
175.9521181694177,2723.471741630537,-175.9521181694177]},
{"from":66,"to":89,"points":[6491.796625087424,1917.7993724147564,6491.796625087424,1927.7993
724147564,6491.796625087424,2037.4263782371127,6526.45217551837,2037.4263782371127,6526.
45217551837,2147.053384059469,6526.45217551837,2157.053384059469]},
{"from":89,"to":86,"points":[6647.563125225401,2185.3098507342734,6657.563125225401,2185.3098
507342734,6671.932323664462,2185.3098507342734,6671.932323664462,2182.446371487979,6686.
301522103525,2182.446371487979,6696.301522103525,2182.446371487979]},
{"from":56,"to":-
131,"points":[6296.761208346462,1844.5624005938726,6296.761208346462,1834.5624005938726,62
96.761208346462,1834.5624005938726,6226.670968951111,1834.5624005938726,6226.67096895111
1,-522.696324602184,5972.976352619178,-522.696324602184,5972.976352619178,-
952.8873662420615,5972.976352619178,-962.8873662420615]},
{"from":-131,"to":58,"points":[5922.002557046262,-962.8873662420614,5922.002557046262,-
944.8873662420614,5922.002557046262,-948,5922.002557046262,-948,5922.002557046262,-
844,6110.200286362687,-
844,6110.200286362687,1833.1882462883614,6110.200286362687,1851.1882462883614]},
{"from":-123,"to":58,"points":[5932.3720994860405,-438.1247020095716,5932.3720994860405,-
428.1247020095716,5932.3720994860405,706.5317721393949,6072.853763077775,706.53177213939
49,6072.853763077775,1841.1882462883614,6072.853763077775,1851.1882462883614]},
{"from":-132,"to":-133,"points":[6137.586703944437,-1438.0392373915888,6137.586703944437,-
1428.0392373915888,6137.586703944437,-1192.302508907023,6137.586703944437,-
1192.302508907023,6137.586703944437,-956.565780422457,6137.586703944437,-
946.565780422457]},
{"from":-133,"to":58,"points":[6137.586703944437,-857.5399137232381,6137.586703944437,-
847.5399137232381,6137.586703944437,488.8241662825617,6147.546809647599,488.824166282561
7,6147.546809647599,1825.1882462883614,6147.546809647599,1851.1882462883614]},
{"from":130,"to":-
120,"points":[4695.980675204417,2235.625592013711,4705.980675204417,2235.625592013711,4777.
904924675019,2235.625592013711,4777.904924675019,2234.905647360726,4849.829174145621,223
4.905647360726,4859.829174145621,2234.905647360726]},
{"from":126,"to":61,"points":[4789.601445570509,-1667.1904468265689,4789.601445570509,-
1657.1904468265689,4789.601445570509,-1657.1904468265689,6236.000076342599,-
1657.1904468265689,6236.000076342599,-1007.4002995916708,6329.558552283851,-
1007.4002995916708,6339.558552283851,-1007.4002995916708]},
{"from":46,"to":-136,"points":[5545.560116664124,-1448.2220889791238,5545.560116664124,-
1430.2220889791238,5545.560116664124,-1298.287280866944,5576.222462308365,-
1298.287280866944,5576.222462308365,-1166.3524727547642,5576.222462308365,-
1156.3524727547642]},
{"from":-136,"to":-138,"points":[5548.985045723079,-969.7878060067177,5548.985045723079,-
959.7878060067177,5548.985045723079,187.05287778218667,5443.5613432907085,187.0528777821
8667,5443.5613432907085,1333.8935615710911,5443.5613432907085,1343.8935615710911]},
{"from":-136,"to":-139,"points":[5603.459878893651,-969.7878060067177,5603.459878893651,-
959.7878060067177,5603.459878893651,180.6091848719792,5781.258809370965,180.609184871979
2,5781.258809370965,1321.006175750676,5781.258809370965,1331.006175750676]},

{"from":-
138,"to":23,"points":[5443.5613432907085,1384.1500282458956,5443.5613432907085,1394.15002824
58956,5443.5613432907085,1396,5443.5613432907085,1396,5443.5613432907085,1916,5588.226501
2161415,1916,5588.2265012161415,2182.45531874438,5588.2265012161415,2200.45531874438]},
{"from":-
139,"to":23,"points":[5781.258809370965,1403.7755757750901,5781.258809370965,1413.7755757750
901,5781.258809370965,1412,5781.258809370965,1412,5781.258809370965,1916,5660.91456884309
45,1916,5660.9145688430945,2190.45531874438,5660.9145688430945,2200.45531874438]},
{"from":-143,"to":-142,"points":[3274.112817548195,-1642.8057468143625,3274.112817548195,-
1632.8057468143625,3274.112817548195,-1630.836620451671,3420.0000763425983,-
1630.836620451671,3420.0000763425983,-758.836620451671,3463.3603643063593,-
758.836620451671,3463.3603643063593,-511.9912102957874,3463.3603643063593,-
501.9912102957874]},
{"from":-137,"to":-
135,"points":[3004.643358664798,1458.8190869026355,3004.643358664798,1476.8190869026355,30
04.643358664798,1476.8190869026355,2972,1476.8190869026355,2972,1852,3091.812734060736,18
52,3091.812734060736,2173.083445957115,3091.812734060736,2183.083445957115]},
{"from":-146,"to":-
145,"points":[2946.019890307112,767.2049311067325,2956.019890307112,767.2049311067325,2962.
330619288664,767.2049311067325,2962.330619288664,766.1334254666173,2968.6413482702155,76
6.1334254666173,2978.6413482702155,766.1334254666173]},
{"from":-149,"to":-150,"points":[3062.4415948614273,-930.3093137476519,3062.4415948614273,-
920.3093137476519,3062.4415948614273,-918.8366204516709,3028.0000763425983,-
918.8366204516709,3028.0000763425983,-814.8366204516709,3161.791930248479,-
814.8366204516709,3161.791930248479,723.4724553504626,3161.791930248479,733.472455350462
6]},
{"from":-150,"to":-
128,"points":[3198.274622966985,733.4724553504626,3198.274622966985,715.4724553504626,3198.
274622966985,713.1633795483291,3204.0000763425987,713.1633795483291,3204.0000763425987,-
878.8366204516709,3276.059027674045,-878.8366204516709,3276.059027674045,-
920.3093137476521,3276.059027674045,-930.3093137476521]},
{"from":-155,"to":-156,"points":[3751.447257123919,-1423.1166873018024,3751.447257123919,-
1397.1166873018024,3751.447257123919,-1205.097700536934,3738.559143998786,-
1205.097700536934,3738.559143998786,-1013.0787137720662,3738.559143998786,-
1003.0787137720662]},
{"from":-155,"to":-150,"points":[3705.5757590160088,-1423.1166873018028,3705.5757590160088,-
1405.1166873018028,3705.5757590160088,-1404,3444,-1404,3444,-764,3404,-
764,3404,252,3234.757315685491,252,3234.757315685491,723.4724553504626,3234.757315685491,7
33.4724553504626]},
{"from":-160,"to":-157,"points":[4044.7176437297544,-1650.9339801517642,4044.7176437297544,-
1640.9339801517642,4044.7176437297544,-1640.9339801517642,4084.0000763425987,-
1640.9339801517642,4084.0000763425987,-433.7751215632261,4012.2336031646146,-
433.7751215632261,4012.2336031646146,-420.53016404118756,4012.2336031646146,-
394.53016404118756]},
{"from":-158,"to":-157,"points":[3943.7686040346425,-1436.1605983997115,3943.7686040346425,-
1426.1605983997115,3943.7686040346425,-1425.775121563226,3900.0000763425983,-
1425.775121563226,3900.0000763425983,-433.7751215632262,3982.270771031639,-

433.7751215632262,3982.270771031639,-412.5301640411877,3982.270771031639,-
394.5301640411877]},
{"from":-161,"to":-157,"points":[4328.543097589042,-1650.9339801517644,4328.543097589042,-
1640.9339801517644,4328.543097589042,-1640.318548832985,4143.335484475555,-
1640.318548832985,4143.335484475555,-432.318548832985,4050.7572444784396,-
432.318548832985,4050.7572444784396,-412.53016404118756,4050.7572444784396,-
394.53016404118756]},
{"from":-159,"to":-157,"points":[3701.0340948924736,-1650.9339801517647,3701.0340948924736,-
1640.9339801517647,3701.0340948924736,-1640.9339801517647,3810.670968951111,-
1640.9339801517647,3810.670968951111,-1426.696324602184,3842.670968951111,-
1426.696324602184,3842.670968951111,-434.69632460218395,3948.0275343082385,-
434.69632460218395,3948.0275343082385,-404.5301640411877,3948.0275343082385,-
394.5301640411877]},
{"from":-160,"to":-162,"points":[4104.6697006145205,-1650.9339801517642,4104.6697006145205,-
1640.9339801517642,4104.6697006145205,-1076.4625952237757,4231.447399529949,-
1076.4625952237757,4231.447399529949,-511.9912102957872,4231.447399529949,-
501.9912102957872]},
{"from":-162,"to":-163,"points":[4231.447399529948,-429.2218102713733,4231.447399529948,-
419.2218102713733,4231.447399529948,-369.78654829167465,4231.447399529948,-
369.78654829167465,4231.447399529948,-320.3512863119761,4231.447399529948,-
310.3512863119761]},
{"from":-157,"to":-165,"points":[3922.3451067656883,-366.273697366383,3912.3451067656883,-
366.273697366383,3875.288810562901,-366.273697366383,3875.288810562901,-
358.14546402898065,3838.232514360114,-358.14546402898065,3828.232514360114,-
358.14546402898065]},
{"from":-156,"to":-169,"points":[3768.450267452562,-930.309313747652,3768.450267452562,-
912.309313747652,3768.450267452562,-708.0861453530185,3739.997994401618,-
708.0861453530185,3739.997994401618,-503.86297695838493,3739.997994401618,-
493.86297695838493]},
{"from":-169,"to":-151,"points":[3709.627254146898,-437.3500436087756,3709.627254146898,-
427.3500436087756,3709.627254146898,-424.31854883298513,3709.627254146898,-
424.31854883298513,3709.627254146898,-416.31854883298513,3505.7119268063598,-
416.31854883298513,3505.7119268063598,192.84543432673445,3505.7119268063598,210.84543432
673445]},
{"from":-157,"to":-151,"points":[3982.270771031639,-338.01723069157833,3982.270771031639,-
328.01723069157833,3982.270771031639,-63.58589818242194,3537.4755986813593,-
63.58589818242194,3537.4755986813593,200.84543432673445,3537.4755986813593,210.845434326
73445]},
{"from":-157,"to":-162,"points":[4076.4396720209897,-394.5301640411877,4076.4396720209897,-
404.5301640411877,4076.4396720209897,-406.8366204516709,4084.0000763425983,-
406.8366204516709,4084.0000763425983,-453.47827694617797,4147.215763482585,-
453.47827694617797,4157.215763482585,-453.47827694617797]},
{"from":-163,"to":-170,"points":[4144.713009271159,-273.96658629976906,4134.713009271159,-
273.96658629976906,3005.412767385761,-273.96658629976906,3005.412767385761,-
828.516080610233,1876.112525500364,-828.516080610233,1866.112525500364,-
828.516080610233]},

{"from":-165,"to":-138,"points":[3744.990479448005,-321.7607640167736,3744.990479448005,-311.7607640167736,3744.990479448005,511.0663987771587,5383.829185901388,511.0663987771587,5383.829185901388,1333.893561571091,5383.829185901388,1343.893561571091]},
{"from":-138,"to":-153,"points":[5383.829185901386,1384.1500282458956,5383.829185901386,1394.1500282458956,5383.829185901386,1396,5383.829185901386,1396,5383.829185901386,1740,3028,1740,3028,2225.527389461281,2956.282367934983,2225.527389461281,2946.282367934983,2225.527389461281]},
{"from":-152,"to":127,"points":[6358.525049343419,-1485.0995337682596,6348.525049343419,-1485.0995337682596,6348.525049343419,-1485.0995337682596,6348.525049343419,-1424.3185488329852,4711.335484475555,-1424.3185488329852,4711.335484475555,-974.8222470972617,4620.089314432037,-974.8222470972617,4610.089314432037,-974.8222470972617]},
{"from":-152,"to":61,"points":[6443.711732998204,-1440.5866004186498,6443.711732998204,-1430.58660041865,6443.711732998204,-1246.2499166799653,6423.502064246742,-1246.2499166799653,6423.502064246742,-1061.9132329412805,6423.502064246742,-1051.9132329412805]},
{"from":-145,"to":-150,"points":[3099.2440704381847,766.1334254666173,3109.2440704381847,766.1334254666173,3112.276653984079,766.1334254666173,3112.276653984079,769.8571553626697,3115.3092375299734,769.8571553626697,3125.3092375299734,769.8571553626697]},
{"from":-127,"to":26,"points":[2576.606751396162,-228.59328485642942,2576.606751396162,-238.59328485642942,2576.606751396162,-276.6707084064431,2469.1922982883693,-276.6707084064431,2469.1922982883693,-314.7481319564568,2469.1922982883693,-324.7481319564568]},
{"from":33,"to":-148,"points":[2704.145535993898,-946.5657804224569,2704.145535993898,-928.5657804224569,2704.145535993898,-928.3185488329851,2704.145535993898,-928.3185488329851,2704.145535993898,-920.3185488329851,3002.0655483428277,-920.3185488329851,3002.0655483428277,193.28664649072596,3002.0655483428277,203.28664649072596]},
{"from":34,"to":-148,"points":[3951.9891963249997,-445.0182537151528,3951.9891963249997,-435.0182537151528,3951.9891963249997,-432.31854883298513,3951.9891963249997,-432.31854883298513,3951.9891963249997,-416.31854883298513,3057.2362585885962,-416.31854883298513,3057.2362585885962,193.28664649072607,3057.2362585885962,203.28664649072607]},
{"from":-157,"to":34,"points":[4042.1964352975897,-394.5301640411877,4042.1964352975897,-428.5301640411877,4036.5655865022127,-428.5301640411877,4036.5655865022127,-419.0182537151528,4030.934737706835,-419.0182537151528,4030.934737706835,-445.0182537151528]},
{"from":38,"to":83,"points":[5315.189014740736,-1667.1904468265689,5315.189014740736,-1657.1904468265689,5315.189014740736,-1656.318548832985,5013.1632114729355,-1656.318548832985,5013.1632114729355,-1021.2069471094684,5013.1632114729355,-1011.2069471094684]},
{"from":-162,"to":-170,"points":[4157.215763482584,-477.7347436209826,4147.215763482584,-477.7347436209826,4146.670968951111,-477.7347436209826,4146.670968951111,-530.696324602184,3026.670968951111,-530.696324602184,3026.670968951111,-852.7725472850375,1884.1125255003635,-852.7725472850375,1866.1125255003635,-852.7725472850375]},

{"from":33,"to":25,"points":[2613.2707877639177,-946.5657804224569,2613.2707877639177,-
936.5657804224569,2613.2707877639177,-721.6748557094064,2473.8258015946317,-
721.6748557094064,2473.8258015946317,-506.7839309963559,2473.8258015946317,-
496.7839309963559]},
{"from":-143,"to":-128,"points":[3233.159749768654,-1642.8057468143622,3233.159749768654,-
1624.8057468143622,3233.159749768654,-1318.9422302932144,3304.5998449347867,-
1318.9422302932144,3304.5998449347867,-1013.0787137720663,3304.5998449347867,-
1003.0787137720663]},
{"from":-148,"to":-
146,"points":[3029.6509034657115,276.0560465151401,3029.6509034657115,286.0560465151401,30
29.6509034657115,507.502255473534,2854.52098131053,507.502255473534,2854.52098131053,728.
9484644319278,2854.52098131053,738.9484644319278]},
{"from":-143,"to":-149,"points":[3192.206681989113,-1642.8057468143622,3192.206681989113,-
1632.8057468143622,3192.206681989113,-1322.9422302932144,3062.4415948614273,-
1322.9422302932144,3062.4415948614273,-1013.0787137720663,3062.4415948614273,-
1003.0787137720663]},
{"from":35,"to":-
137,"points":[3058.416880588029,1379.6297308058176,3058.416880588029,1397.6297308058176,30
58.416880588029,1403.0961755168244,3033.6347196470574,1403.0961755168244,3033.6347196470
574,1408.5626202278308,3033.6347196470574,1418.5626202278308]},
{"from":-
150,"to":35,"points":[3198.274622966985,806.2418553748766,3198.274622966985,816.241855374876
6,3198.274622966985,1044.4228597407377,3002.5605726046306,1044.4228597407377,3002.560572
6046306,1272.6038641065988,3002.5605726046306,1290.6038641065988]},
{"from":-118,"to":-170,"points":[1920.1000022744572,-914.0528470728474,1920.1000022744572,-
904.0528470728475,1920.1000022744572,-895.5409305163448,1806.3710755085015,-
895.5409305163448,1806.3710755085015,-887.0290139598422,1806.3710755085015,-
877.0290139598422]},
{"from":12,"to":-170,"points":[1730.0090062014103,-946.5657804224569,1730.0090062014103,-
936.5657804224569,1730.0090062014103,-911.7973971911496,1746.6296255166396,-
911.7973971911496,1746.6296255166396,-887.0290139598422,1746.6296255166396,-
877.0290139598422]},
{"from":2,"to":-167,"points":[1519.008883371618,-946.5657804224568,1519.008883371618,-
936.5657804224568,1519.008883371618,-903.6691638537471,1590.2982610136032,-
903.6691638537471,1590.2982610136032,-870.7725472850375,1590.2982610136032,-
860.7725472850375]},
{"from":-164,"to":13,"points":[1702.458266380114,-689.538482833184,1702.458266380114,-
679.538482833184,1702.458266380114,-577.913406677847,1802.5534497441356,-
577.913406677847,1802.5534497441356,-476.28833052251,1802.5534497441356,-
466.28833052251]},
{"from":-164,"to":3,"points":[1642.7720430321322,-689.538482833184,1642.7720430321322,-
679.538482833184,1642.7720430321322,-577.583579785241,1567.9280932912527,-
577.583579785241,1567.9280932912527,-475.6286767372979,1567.9280932912527,-
465.6286767372979]},
{"from":123,"to":-
173,"points":[2182.6997749883267,1381.7600951103668,2182.6997749883267,1391.7600951103668,
2182.6997749883267,1575.7060755867838,2089.7530646767077,1575.7060755867838,2089.7530646
767077,1759.6520560632007,2089.7530646767077,1769.6520560632007]},

{"from":-169,"to":-147,"points":[3770.3687346563383,-437.35004360877565,3770.3687346563383,-419.35004360877565,3770.3687346563383,-418.69632460218395,3650.670968951111,-418.69632460218395,3650.670968951111,309.30367539781605,3544.4927967613785,309.30367539781605,3544.4927967613785,1146.6929521225363,3544.4927967613785,1164.6929521225363]},
{"from":-129,"to":-175,"points":[3311.6077208185366,245.88924099023745,3311.6077208185366,255.88924099023745,3311.6077208185366,705.4686249998895,3407.0605732243484,705.4686249998895,3407.0605732243484,1155.0480090095414,3407.0605732243484,1181.0480090095414]},
{"from":-151,"to":-147,"points":[3489.8300908688593,299.8713010259532,3489.8300908688593,309.8713010259532,3489.8300908688593,732.2821265742448,3499.686709267482,732.2821265742448,3499.686709267482,1154.6929521225363,3499.686709267482,1164.6929521225363]},
{"from":-157,"to":-147,"points":[4042.1964352975892,-338.01723069157833,4042.1964352975892,-320.01723069157833,4042.1964352975892,417.337860715479,3589.298884255275,417.337860715479,3589.298884255275,1154.6929521225363,3589.298884255275,1164.6929521225363]},
{"from":88,"to":-176,"points":[6512.343474532491,-459.40397783126195,6512.343474532491,-449.40397783126195,6512.343474532491,142.5049422281843,6519.955598794646,142.5049422281843,6519.955598794646,734.4138622876305,6519.955598794646,744.4138622876305]},
{"from":85,"to":-176,"points":[6932.314379703115,2141.411209623385,6932.314379703115,2131.411209623385,6932.314379703115,2132,6932.314379703115,2132,6932.314379703115,1324,6504.345483743377,1324,6504.345483743377,810.6703289624352,6504.345483743377,784.6703289624352]},
{"from":64,"to":-176,"points":[6344.528041985519,1322.8779424132738,6344.528041985519,1312.8779424132738,6344.528041985519,1053.7741356878546,6473.125253640837,1053.7741356878546,6473.125253640837,794.6703289624352,6473.125253640837,784.6703289624352]},
{"from":25,"to":-175,"points":[2473.8258015946312,-440.2709976467464,2473.8258015946312,-430.2709976467464,2473.8258015946312,-430.0829020928786,2511.3354844755554,-430.0829020928786,2511.3354844755554,297.9170979071214,3368.0352855961746,297.9170979071214,3368.0352855961746,1163.0480090095414,3368.0352855961746,1181.0480090095414]},
{"from":26,"to":-175,"points":[2439.3852113437893,-324.74813195645686,2439.3852113437893,-306.74813195645686,2439.3852113437893,432.1499385265422,3329.009997968001,432.1499385265422,3329.009997968001,1171.0480090095414,3329.009997968001,1181.0480090095414]},
{"from":-142,"to":-180,"points":[3516.2998174313593,-501.9912102957873,3516.2998174313593,-519.9912102957874,3516.2998174313593,-651.7719662306696,3499.0933414702527,-651.7719662306696,3499.0933414702527,-783.5527221655518,3499.0933414702527,-793.5527221655518]},
{"from":-128,"to":-180,"points":[3333.140662195529,-930.309313747652,3333.140662195529,-920.309313747652,3333.140662195529,-898.315717968809,3499.093341470253,-898.315717968809,3499.093341470253,-876.3221221899659,3499.093341470253,-866.3221221899659]},
{"from":-149,"to":-180,"points":[3151.253614209572,-966.6940137598589,3161.253614209572,-966.6940137598589,3164.0000763425983,-966.6940137598589,3164.0000763425983,-918.8366204516709,3412.0000763425983,-918.8366204516709,3412.0000763425983,-842.0656555151612,3444.287253976356,-842.0656555151612,3454.287253976356,-842.0656555151612]},
{"from":-155,"to":-180,"points":[3659.7042609080986,-1423.1166873018024,3659.7042609080986,-1413.1166873018024,3659.7042609080986,-1148.719404745884,3543.8994289641496,-

1148.719404745884,3543.8994289641496,-884.322122189966,3543.8994289641496,-
866.322122189966]},
{"from":-158,"to":-180,"points":[3893.107069407364,-1436.1605983997115,3893.107069407364,-
1426.1605983997115,3893.107069407364,-1151.2413602948386,3588.7055164580456,-
1151.2413602948386,3588.7055164580456,-876.322122189966,3588.7055164580456,-
866.322122189966]},
{"from":-156,"to":-180,"points":[3708.66802054501,-930.309313747652,3708.66802054501,-
920.309313747652,3708.66802054501,-898.315717968809,3597.6667339568253,-
898.315717968809,3597.6667339568253,-876.3221221899659,3597.6667339568253,-
866.3221221899659]},
{"from":34,"to":-180,"points":[3991.4619670159177,-517.7876537395668,3991.4619670159177,-
527.7876537395668,3991.4619670159177,-655.6701879525593,3588.705516458046,-
655.6701879525593,3588.705516458046,-783.5527221655518,3588.705516458046,-
793.5527221655518]},
{"from":26,"to":-181,"points":[2459.256602640176,-365.0045986312615,2459.256602640176,-
375.0045986312615,2459.256602640176,-375.0045986312615,2548.0000763425983,-
375.0045986312615,2548.0000763425983,-510.8366204516709,3116.2348978925215,-
510.8366204516709,3116.2348978925215,-816.0656555151612,3116.2348978925215,-
826.0656555151612]},
{"from":27,"to":35,"points":[2552.469401670238,1339.9739680806995,2562.469401670238,1339.9739
680806995,2749.586833145735,1339.9739680806995,2749.586833145735,1335.1167974562084,2936
.704264621232,1335.1167974562084,2946.704264621232,1335.1167974562084]},
{"from":35,"to":-
153,"points":[3030.48872659633,1379.6297308058176,3030.48872659633,1397.6297308058176,3030.
48872659633,1397.6297308058176,2894.015074640712,1397.6297308058176,2894.015074640712,21
87.270922786476,2894.015074640712,2197.270922786476]},
{"from":27,"to":-
153,"points":[2461.4227479226793,1376.3586680929066,2461.4227479226793,1386.3586680929066,
2461.4227479226793,1388,2841.7477813464416,1388,2841.7477813464416,2187.270922786476,284
1.7477813464416,2197.270922786476]},
{"from":-127,"to":-182,"points":[2631.6811227340527,-123.31095148240598,2631.6811227340527,-
113.31095148240598,2631.6811227340527,108.01865443764484,2628.4156274337597,108.01865443
764484,2628.4156274337597,329.3482603576957,2628.4156274337597,339.3482603576957]},
{"from":24,"to":-183,"points":[1990.081027708156,-1650.9339801517642,1990.081027708156,-
1632.9339801517642,1990.081027708156,-1632.9339801517642,2004,-1632.9339801517642,2004,-
396,1892.8648243958062,-
396,1892.8648243958062,2150.829709983095,1892.8648243958062,2200.829709983095]},
{"from":-182,"to":-
183,"points":[2598.9072529016958,412.11766038210976,2598.9072529016958,422.11766038210976,
2598.9072529016958,1290.4736851826024,1918.017401387296,1290.4736851826024,1918.01740138
7296,2158.829709983095,1918.017401387296,2200.829709983095]},
{"from":24,"to":-184,"points":[2014.408455163513,-1650.9339801517642,2014.408455163513,-
1616.9339801517642,2014.408455163513,-1620,2014.408455163513,-
1620,2014.408455163513,1388,2180,1388,2180,1836,2446.186346198313,1836,2446.186346198313,2
182.7014766456928,2446.186346198313,2192.7014766456928]},
{"from":-182,"to":-
184,"points":[2657.9240019658237,412.11766038210976,2657.9240019658237,430.11766038210976,

2657.9240019658237,428,2657.9240019658237,428,2657.9240019658237,1836,2490.744306708567,1
836,2490.744306708567,2174.7014766456928,2490.744306708567,2192.7014766456928]},
{"from":62,"to":64,"points":[6366.653081790085,264.62445811267537,6366.653081790085,274.62445
811267537,6366.653081790085,789.7512002629746,6289.52031595199,789.7512002629746,6289.52
031595199,1304.8779424132738,6289.52031595199,1322.8779424132738]},
{"from":-137,"to":-
186,"points":[3062.6260806293167,1458.8190869026355,3062.6260806293167,1476.8190869026355,
3062.6260806293167,1568.1245254054857,3056.7661550090897,1568.1245254054857,3056.7661550
090897,1659.4299639083358,3056.7661550090897,1669.4299639083358]},
{"from":-185,"to":-
187,"points":[6711.2184682097195,2296.87123672408,6693.2184682097195,2296.87123672408,6692,
2296.87123672408,6692,2296.87123672408,6684,2296.87123672408,6684,1690.6416931271463,3337.
6622752679054,1690.6416931271463,3327.6622752679054,1690.6416931271463]},
{"from":89,"to":-
185,"points":[6571.868781658506,2213.5663174090782,6571.868781658506,2223.5663174090782,65
71.868781658506,2266.550653380574,6636.543624934113,2266.550653380574,6701.2184682097195
,2266.550653380574,6711.2184682097195,2266.550653380574]},
{"from":86,"to":-
185,"points":[6786.069451180672,2210.702838162783,6786.069451180672,2220.702838162783,6786.
069451180672,2225.5305707686266,6819.041283151125,2225.5305707686266,6819.041283151125,2
230.3583033744703,6819.041283151125,2248.3583033744703]},
{"from":85,"to":-
185,"points":[6985.6229597202055,2214.180609647799,6985.6229597202055,2224.180609647799,69
85.6229597202055,2284.7430033866776,6934.287825171017,2284.7430033866776,6882.9526906218
28,2284.7430033866776,6872.952690621828,2284.7430033866776]},
{"from":-185,"to":-
121,"points":[6711.2184682097195,2272.6147700492756,6701.2184682097195,2272.6147700492756,
6700,2272.6147700492756,6700,2272.6147700492756,6484,2272.6147700492756,6484,2156,5516,215
6,5516,1870.163585463343,5260.35147624619,1870.163585463343,5250.35147624619,1870.1635854
63343]},
{"from":-185,"to":-
119,"points":[6775.912157174564,2248.358303374471,6775.912157174564,2230.358303374471,6775.
912157174564,2228,6484,2228,6484,2156,5004.041638673725,2156,5004.041638673725,1418.225692
5836793,5004.041638673725,1408.2256925836793]},
{"from":-167,"to":-164,"points":[1590.2982610136032,-820.5160806102327,1590.2982610136032,-
810.5160806102327,1590.2982610136032,-799.5402150713177,1672.6151547061231,-
799.5402150713177,1672.6151547061231,-788.5643495324027,1672.6151547061231,-
778.5643495324027]},
{"from":-170,"to":-164,"points":[1776.5003505125705,-804.2596139354282,1776.5003505125705,-
794.2596139354282,1776.5003505125705,-791.4119817339155,1702.458266380114,-
791.4119817339155,1702.458266380114,-788.5643495324027,1702.458266380114,-
778.5643495324028]},
{"from":-189,"to":-
109,"points":[2283.634752823567,1829.2518795669096,2283.634752823567,1839.2518795669096,22
83.634752823567,1845.0486726259269,2283.634752823567,1845.0486726259269,2283.63475282356
7,1850.8454656849442,2283.634752823567,1860.8454656849442]},
{"from":-173,"to":-
189,"points":[2167.8036399330554,1789.780289400603,2177.8036399330554,1789.780289400603,21

80.954528867325,1789.780289400603,2180.954528867325,1784.7389462173003,2184.105417801594
3,1784.7389462173003,2194.1054178015943,1784.7389462173003]},
{"from":-172,"to":-
189,"points":[2407.3068467045996,1792.8671795547025,2397.3068467045996,1792.8671795547025,
2390.23546727507,1792.8671795547025,2390.23546727507,1784.7389462173003,2383.16408784553
96,1784.7389462173003,2373.1640878455396,1784.7389462173003]},
{"from":-187,"to":-
171,"points":[3238.0501002801125,1727.0263931393533,3238.0501002801125,1737.0263931393533,
3238.0501002801125,1741.7544363409243,3163.770340359156,1741.7544363409243,3163.77034035
9156,1746.4824795424954,3163.770340359156,1756.4824795424954]},
{"from":-186,"to":-
171,"points":[3056.7661550090897,1709.6864305831405,3056.7661550090897,1719.6864305831405,
3056.7661550090897,1733.084455062818,3104.0841170111744,1733.084455062818,3104.084117011
1744,1746.4824795424954,3104.0841170111744,1756.4824795424954]},
{"from":-171,"to":-
135,"points":[3133.927228685165,1845.5083462417142,3133.927228685165,1855.5083462417142,31
33.927228685165,2010.2958960994147,3147.7603760692,2010.2958960994147,3147.7603760692,21
65.083445957115,3147.7603760692,2183.083445957115]},
{"from":-175,"to":-
188,"points":[3368.0352855961746,1221.304475684346,3368.0352855961746,1231.304475684346,33
68.0352855961746,1246.523639702715,3419.5744389656006,1246.523639702715,3419.57443896560
06,1261.7428037210839,3419.5744389656006,1271.7428037210839]},
{"from":-147,"to":-
188,"points":[3551.897485339335,1240.2693889159602,3551.897485339335,1250.2693889159602,35
51.897485339335,1257.4096147030268,3486.6653508915388,1257.4096147030268,3486.6653508915
388,1264.5498404900936,3486.6653508915388,1274.5498404900936]},
{"from":-
188,"to":35,"points":[3359.8882156176187,1316.2557370706932,3349.8882156176187,1316.25573707
06932,3237.080702094523,1316.2557370706932,3237.080702094523,1335.1167974562081,3124.273
1885714275,1335.1167974562081,3114.2731885714275,1335.1167974562081]},
{"from":-174,"to":-151,"points":[3341.4508324925273,-777.2962554907473,3341.4508324925273,-
767.2962554907473,3341.4508324925273,-283.2254105820064,3442.1845830563593,-
283.2254105820064,3442.1845830563593,200.8454343267344,3442.1845830563593,210.8454343267
3433]},
{"from":-174,"to":-129,"points":[3281.764609144546,-777.2962554907473,3281.764609144546,-
759.2962554907473,3281.764609144546,-302.08820726246194,3339.939998752293,-
302.08820726246194,3339.939998752293,155.11984096582336,3339.939998752293,173.1198409658
2336]},
{"from":-180,"to":-174,"points":[3454.287253976356,-817.8091888403565,3436.287253976356,-
817.8091888403565,3423.7121549084327,-817.8091888403565,3423.7121549084327,-
821.8091888403566,3411.137055840509,-821.8091888403566,3401.137055840509,-
821.8091888403566]},
{"from":-181,"to":-174,"points":[3194.285473148869,-846.1938888525635,3204.285473148869,-
846.1938888525635,3208.181929472717,-846.1938888525635,3208.181929472717,-
821.8091888403566,3212.0783857965644,-821.8091888403566,3222.0783857965644,-
821.8091888403566]},
{"from":-
168,"to":89,"points":[6596.175381934909,920.0307426226954,6596.175381934909,930.030742622695

4,6596.175381934909,932,6596.175381934909,932,6596.175381934909,940,6660,940,6660,1924,6556
.729912945128,1924,6556.729912945128,2131.053384059469,6556.729912945128,2157.0533840594
69]},
{"from":-
168,"to":86,"points":[6655.861605282891,920.0307426226954,6655.861605282891,930.030742622695
4,6655.861605282891,932,6741.185486642098,932,6741.185486642098,2144.1899048131736,6741.18
5486642098,2154.1899048131736]},
{"from":-176,"to":-
168,"points":[6545.972457213429,784.6703289624353,6545.972457213429,794.670328962435,6545.9
72457213429,807.8376024429551,6596.175381934908,807.8376024429551,6596.175381934908,821.
0048759234753,6596.175381934908,831.004875923475]},
{"from":-178,"to":-
168,"points":[6732.372145772143,792.7099136802375,6732.372145772143,802.7099136802372,6732.
372145772143,811.8573948018562,6655.86160528289,811.8573948018562,6655.86160528289,821.0
048759234753,6655.86160528289,831.004875923475]},
{"from":126,"to":87,"points":[4848.197698011915,-1703.5751468387757,4858.197698011915,-
1703.5751468387757,4860.000076342599,-1703.5751468387757,4860.000076342599,-
1265.7751215632259,6516.000076342599,-1265.7751215632259,6516.000076342599,-
1010.554332188513,6542.15881099815,-1010.554332188513,6552.15881099815,-
1010.554332188513]},
{"from":-190,"to":-
177,"points":[7278.968432179319,276.26143067836665,7278.968432179319,286.26143067836665,72
78.968432179319,803.6338032145213,7146.6486503923115,803.6338032145213,7146.648650392311
5,1321.006175750676,7146.6486503923115,1331.006175750676]},
{"from":-190,"to":-
192,"points":[7337.694669158485,276.26143067836665,7337.694669158485,286.26143067836665,73
37.694669158485,576.3499763946655,7360.666659607288,576.3499763946655,7360.666659607288,
866.4385221109626,7360.666659607288,876.4385221109626]},
{"from":-191,"to":-
190,"points":[6990.952364182142,65.284420048176,7000.952364182142,65.284420048176,7000.9523
64182142,65.284420048176,7000.952364182142,135.68145116701487,7255.477937387653,135.6814
5116701487,7255.477937387653,193.4920306539526,7255.477937387653,203.4920306539526]},
{"from":-193,"to":-
190,"points":[7138.048623943744,123.78101370581601,7138.048623943744,133.781013705816,7138.
048623943744,159.6365221798843,7290.713679575152,159.6365221798843,7290.713679575152,185
.4920306539526,7290.713679575152,203.4920306539526]},
{"from":-196,"to":-
190,"points":[7280.954612802691,81.91402533827261,7280.954612802691,99.91402533827261,7280.
954612802691,138.7030279961126,7325.9494217626525,138.7030279961126,7325.9494217626525,1
77.4920306539526,7325.9494217626525,203.4920306539526]},
{"from":-195,"to":-
190,"points":[7497.075737173839,62.87987758884617,7497.075737173839,72.87987758884617,7497.
075737173839,133.1859541213994,7361.185163950152,133.1859541213994,7361.185163950152,193
.4920306539526,7361.185163950152,203.4920306539526]},
{"from":-
177,"to":65,"points":[7042.423868084369,1367.390875762883,7032.423868084369,1367.39087576288
3,6846.661238892662,1367.390875762883,6846.661238892662,1367.390875762883,6660.898609700
956,1367.390875762883,6650.898609700956,1367.390875762883]},

{"from":-
192,"to":65,"points":[7317.2499085086565,932.9514554605737,7317.2499085086565,942.9514554605
737,7317.2499085086565,1131.9788156056247,6559.050938192167,1131.9788156056247,6559.0509
38192167,1321.006175750676,6559.050938192167,1331.006175750676]},
{"from":-
177,"to":89,"points":[7081.508161449848,1403.7755757750901,7081.508161449848,1413.7755757750
901,7081.508161449848,1780.4144799172796,6617.285387798643,1780.4144799172796,6617.28538
7798643,2147.053384059469,6617.285387798643,2157.053384059469]},
{"from":-
177,"to":86,"points":[7120.592454815326,1403.7755757750901,7120.592454815326,1421.7755757750
901,7120.592454815326,1778.9827402941319,6786.069451180672,1778.9827402941319,6786.06945
1180672,2136.1899048131736,6786.069451180672,2154.1899048131736]},
{"from":-
177,"to":85,"points":[7159.676748180805,1403.7755757750901,7159.676748180805,1429.7755757750
901,7159.676748180805,1776.5933926992375,7003.392486392569,1776.5933926992375,7003.39248
6392569,2123.411209623385,7003.392486392569,2141.411209623385]},
{"from":-
192,"to":89,"points":[7346.194409241079,932.9514554605738,7346.194409241079,950.951455460573
8,7346.194409241079,1545.0024197600214,6587.007650371885,1545.0024197600214,6587.0076503
71885,2139.053384059469,6587.007650371885,2157.053384059469]},
{"from":-
192,"to":86,"points":[7375.1389099735,932.9514554605738,7375.1389099735,958.9514554605738,73
75.1389099735,1551.5706801368738,6830.953415719247,1551.5706801368738,6830.953415719247,
2144.1899048131736,6830.953415719247,2154.1899048131736]},
{"from":-
192,"to":85,"points":[7404.083410705922,932.9514554605738,7404.083410705922,966.951455460573
8,7404.083410705922,1549.1813325419794,7038.931539737296,1549.1813325419794,7038.9315397
37296,2131.411209623385,7038.931539737296,2141.411209623385]},
{"from":83,"to":-178,"points":[5041.591635036575,-938.4375470850541,5041.591635036575,-
928.4375470850541,5041.591635036575,-930.696324602184,5041.591635036575,-
930.696324602184,5041.591635036575,-786.696324602184,6762.242870768074,-
786.696324602184,6762.242870768074,701.9405136558238,6762.242870768074,719.9405136558238
]},
{"from":127,"to":-178,"points":[4556.979852965565,-946.5657804224563,4556.979852965565,-
936.5657804224563,4556.979852965565,-113.31263338331627,6702.5014207762115,-
113.31263338331627,6702.5014207762115,709.9405136558238,6702.5014207762115,719.940513655
8238]},
{"from":-183,"to":-
195,"points":[2043.780286344746,2219.0220599891986,2053.780286344746,2219.0220599891986,20
53.780286344746,2219.0220599891986,2053.780286344746,2140,6724,2140,6724,868,7412,868,7412,
-18.14598911037264,7418.3633195713,-18.14598911037264,7436.3633195713,-
18.14598911037264]},
{"from":-183,"to":-
196,"points":[2018.627709353256,2200.829709983095,2018.627709353256,2190.829709983095,2018.
627709353256,2188,2018.627709353256,2188,2018.627709353256,2180,2724,2180,2724,-
19.49654137315312,7202.242195200152,-19.49654137315312,7220.242195200152,-
19.49654137315312]},

{"from":-183,"to":-
193,"points":[1993.475132361766,2200.829709983095,1993.475132361766,2182.829709983095,1993.
475132361766,63.011613681402,4496.049460550216,63.011613681402,6998.623788738666,63.0116
13681402,7016.623788738666,63.011613681402]},
{"from":-183,"to":-
191,"points":[1968.3225553702762,2200.829709983095,1968.3225553702762,2174.829709983095,19
68.3225553702762,65.284420048176,4381.075867674159,65.284420048176,6793.829179978041,65.2
84420048176,6811.829179978041,65.284420048176]},
{"from":-184,"to":-
195,"points":[2579.8602277290747,2243.573400473818,2613.8602277290747,2243.573400473818,26
12,2243.573400473818,2612,2243.573400473818,2732,2243.573400473818,2732,2148,6724,2148,672
4,868,7412,868,7412,22.366944239236737,7426.3633195713,22.366944239236737,7436.3633195713,
22.366944239236737]},
{"from":-184,"to":-
196,"points":[2579.8602277290747,2230.8554195167862,2605.8602277290747,2230.8554195167862,
2605.8602277290747,2230.8554195167862,2605.8602277290747,2132,7212,2132,7212,31.208741982
559772,7210.242195200152,31.208741982559772,7220.242195200152,31.208741982559772]},
{"from":-184,"to":-
193,"points":[2579.8602277290747,2218.137438559755,2597.8602277290747,2218.137438559755,25
97.8602277290747,2218.137438559755,2597.8602277290747,2132,6996,2132,6996,93.396313693609
03,7006.623788738666,93.39631369360903,7016.623788738666,93.39631369360903]},
{"from":-184,"to":-
191,"points":[2579.8602277290747,2205.419457602724,2589.8602277290747,2205.419457602724,25
88,2205.419457602724,2588,2205.419457602724,2724,2205.419457602724,2724,99.73323672908421,
6801.829179978041,99.73323672908421,6811.829179978041,99.73323672908421]},
{"from":-183,"to":-
197,"points":[1943.169978378786,2200.829709983095,1943.169978378786,2166.829709983095,1943.
169978378786,2164,2756,2164,2756,-540,4761.27216318123,-540,4761.27216318123,-
936.5657804224561,4761.27216318123,-946.5657804224561]},
{"from":-184,"to":-
197,"points":[2535.302267218821,2192.7014766456928,2535.302267218821,2182.7014766456928,25
35.302267218821,2180,2756,2180,2756,-540,4797.710475711748,-540,4797.710475711748,-
928.5657804224561,4797.710475711748,-946.5657804224561]},
{"from":-197,"to":83,"points":[4870.587100772783,-974.8222470972615,4880.587100772783,-
974.8222470972615,4899.232520777401,-974.8222470972615,4899.232520777401,-
974.8222470972615,4917.877940782018,-974.8222470972615,4927.877940782018,-
974.8222470972615]},
{"from":-194,"to":-
197,"points":[7496.818343623527,866.6256038990448,7496.818343623527,856.6256038990448,7496.
818343623527,853.303675397816,7426.670968951111,853.303675397816,7426.670968951111,-
546.696324602184,4834.148788242265,-546.696324602184,4834.148788242265,-
936.5657804224561,4834.148788242265,-946.5657804224561]},
{"from":-191,"to":-
194,"points":[6901.390772080092,134.18205340999242,6901.390772080092,144.18205340999242,69
01.390772080092,496.40382865451863,7525.813618485181,496.40382865451863,7525.81361848518
1,848.6256038990435,7525.813618485181,866.6256038990434]},
{"from":-193,"to":-
194,"points":[7077.336206341205,123.78101370581606,7077.336206341205,141.78101370581606,70

77.336206341205,491.20330880243046,7554.808893346834,491.20330880243046,7554.80889334683
4,840.6256038990435,7554.808893346834,866.6256038990434]},
{"from":-196,"to":-
194,"points":[7341.66703040523,81.91402533827267,7341.66703040523,91.91402533827267,7341.66
703040523,91.91402533827267,7583.804168208488,91.91402533827267,7583.804168208488,832.62
56038990435,7583.804168208488,866.6256038990434]},
{"from":-195,"to":-
194,"points":[7557.788154776378,62.879877588846114,7557.788154776378,72.87987758884611,755
7.788154776378,448.75274074394554,7612.799443070142,448.75274074394554,7612.799443070142
,824.6256038990438,7612.799443070142,866.6256038990434]},
{"from":-199,"to":-
190,"points":[7783.25781625611,41.823410914041425,7773.25781625611,41.823410914041425,7772,
41.823410914041425,7772,76,7612,76,7612,227.74849732875728,7406.420906137652,227.748497328
75728,7396.420906137652,227.74849732875728]},
{"from":-198,"to":-
190,"points":[7618.500572378917,154.16571371802308,7592.500572378917,154.16571371802308,75
03.460739258285,154.16571371802308,7503.460739258285,252.004964003562,7414.420906137652,
252.004964003562,7396.420906137652,252.004964003562]},
{"from":-184,"to":-
198,"points":[2579.8602277290747,2269.00936238788,2629.8602277290747,2269.00936238788,2629.
8602277290747,2269.00936238788,2629.8602277290747,2148,6724,2148,6724,868,7404,868,7404,21
4.93511374243712,7608.500572378917,214.93511374243712,7618.500572378917,214.935113742437
12]},
{"from":-184,"to":-
199,"points":[2579.8602277290747,2256.291381430849,2621.8602277290747,2256.291381430849,26
21.8602277290747,2256.291381430849,2621.8602277290747,2132,7212,2132,7212,116,7628,116,762
8,20.766944239236736,7765.25781625611,20.766944239236736,7783.25781625611,20.76694423923
6736]},
{"from":-183,"to":-
198,"points":[2043.780286344746,2255.406760001406,2069.780286344746,2255.406760001406,2069.
780286344746,2255.406760001406,2069.780286344746,2172,3548,2172,3548,1324,5212,1324,5212,7
32,6460,732,6460,184.55041373023008,7600.500572378917,184.55041373023008,7618.50057237891
7,184.55041373023008]},
{"from":-183,"to":-
199,"points":[2043.780286344746,2237.214409995302,2061.780286344746,2237.214409995302,2061.
780286344746,2237.214409995302,2061.780286344746,2132,7212,2132,7212,116,7628,116,7628,-
0.289522435567946,7757.25781625611,-0.289522435567946,7783.25781625611,-
0.289522435567946]},
{"from":33,"to":-199,"points":[2764.728701480552,-988.9504804346639,2790.728701480552,-
988.9504804346639,2790.728701480552,-988.9504804346639,2790.728701480552,-924,3644,-
924,3644,-788,7628,-788,7628,-21.345989110372642,7773.25781625611,-
21.345989110372642,7783.25781625611,-21.345989110372642]},
{"from":-129,"to":-
196,"points":[3396.604554619806,209.50454097803043,3406.604554619806,209.50454097803043,34
06.604554619806,209.50454097803043,3406.604554619806,-
70.20182472886603,7194.242195200153,-70.20182472886603,7220.242195200153,-
70.20182472886603]},

{"from":33,"to":-191,"points":[2764.728701480552,-960.6940137598591,2774.728701480552,-960.6940137598591,2775.3354844755554,-960.6940137598591,2775.3354844755554,30.835603367267765,6801.829179978042,30.835603367267765,6811.829179978042,30.835603367267765]},
{"from":33,"to":-193,"points":[2764.728701480552,-974.8222470972615,2782.728701480552,-974.8222470972615,2782.728701480552,-974.8222470972615,2782.728701480552,-758.8366204516709,6996.0000763426,-758.8366204516709,6996.0000763426,32.62691366919495,7006.623788738667,32.62691366919495,7016.623788738667,32.62691366919495]},
{"from":-200,"to":-201,"points":[7795.824611409811,-146.52803575614206,7795.824611409811,-136.52803575614206,7795.824611409811,-136.52803575614206,7996,-136.52803575614206,7996,132,7986.719550944455,132,7986.719550944455,922.9514554605735,7986.719550944455,932.9514554605735]},
{"from":-201,"to":-192,"points":[7899.607047892697,953.0796887979759,7889.607047892697,953.0796887979759,7887.335484475556,953.0796887979759,7887.335484475556,953.0796887979759,7455.335484475556,953.0796887979759,7455.335484475556,904.694988785769,7443.027911438344,904.694988785769,7433.027911438344,904.694988785769]},
{"from":-202,"to":-200,"points":[7794.261525788138,-1634.3392658593177,7794.261525788138,-1624.3392658593177,7794.261525788138,-943.0748174947416,7795.824611409811,-943.0748174947416,7795.824611409811,-261.8103691301655,7795.824611409811,-251.8103691301655]},
{"from":-177,"to":-176,"points":[7094.536259238341,1331.006175750676,7094.536259238341,1321.006175750676,7094.536259238341,1317.303675397816,7094.536259238341,1317.303675397816,7094.536259238341,821.303675397816,6535.565713845916,821.303675397816,6535.565713845916,802.6703289624352,6535.565713845916,784.6703289624352]},
{"from":-192,"to":-176,"points":[7288.3054077762345,904.694988785769,7278.3054077762345,904.694988785769,7278.3054077762345,904.694988785769,7278.3054077762345,817.9170979071214,6631.335484475556,817.9170979071214,6631.335484475556,764.5420956250329,6608.006174050995,764.5420956250329,6598.006174050995,764.5420956250329]},
{"from":17,"to":83,"points":[5162.061400401543,-1034.3365009903946,5152.061400401543,-1034.3365009903946,5130.254941282698,-1034.3365009903946,5130.254941282698,-1005.1428304407675,5108.448482163853,-1005.1428304407675,5098.448482163853,-1005.1428304407675]},
{"from":47,"to":83,"points":[5286.390805140686,-924.2398458251661,5276.390805140686,-924.2398458251661,5196.41964365227,-924.2398458251661,5196.41964365227,-950.5657804224567,5116.448482163853,-950.5657804224567,5098.448482163853,-950.5657804224567]},
{"from":-136,"to":83,"points":[5494.510212552505,-1094.164250505415,5476.510212552505,-1094.164250505415,5296.479347358179,-1094.164250505415,5296.479347358179,-999.0787137720661,5116.448482163853,-999.0787137720661,5098.448482163853,-999.0787137720661]},
{"from":51,"to":83,"points":[5667.747370611477,-865.5399137232381,5649.747370611477,-865.5399137232381,5650.670968951111,-865.5399137232381,5650.670968951111,-865.5399137232381,5482.670968951111,-865.5399137232381,5482.670968951111,-970.696324602184,5274.670968951111,-970.696324602184,5274.670968951111,-

956.6298970911577,5124.448482163853,-956.6298970911577,5098.448482163853,-
956.6298970911577]},
{"from":-131,"to":83,"points":[5871.028761473345,-999.2720662542683,5861.028761473345,-
999.2720662542683,5860,-999.2720662542683,5860,-956,5484,-956,5484,-
986.9504804346635,5132.448482163853,-986.9504804346635,5098.448482163853,-
986.9504804346635]},
{"from":-133,"to":83,"points":[6046.4388996841835,-902.0528470728475,6036.4388996841835,-
902.0528470728475,6034.670968951111,-902.0528470728475,6034.670968951111,-
954.696324602184,5482.670968951111,-954.696324602184,5482.670968951111,-
968.75813042856,5140.448482163853,-968.75813042856,5098.448482163853,-968.75813042856]},
{"from":83,"to":-172,"points":[4984.734787909296,-938.4375470850541,4984.734787909296,-
928.4375470850541,4984.734787909296,-928.4375470850541,4964,-
928.4375470850541,4964,820,4884,820,4884,1388,2496.9190216923926,1388,2496.9190216923926,1
746.4824795424954,2496.9190216923926,1756.4824795424954]},
{"from":17,"to":-204,"points":[5212.88835810418,-1014.2082676529923,5212.88835810418,-
1004.2082676529923,5212.88835810418,-926.2562348515664,5206.129413318878,-
926.2562348515664,5206.129413318878,-848.3042020501406,5206.129413318878,-
838.3042020501406]},
{"from":-204,"to":18,"points":[5206.129413318878,-798.0477353753358,5206.129413318878,-
788.0477353753358,5206.129413318878,269.5097663111326,5213.8151634165215,269.50976631113
26,5213.8151634165215,1327.067267997601,5213.8151634165215,1337.067267997601]},
{"from":83,"to":47,"points":[5098.448482163853,-944.5016637537556,5108.448482163853,-
944.5016637537556,5188.41964365227,-944.5016637537556,5188.41964365227,-
889.1457347004917,5268.390805140686,-889.1457347004917,5286.390805140686,-
889.1457347004917]},
{"from":83,"to":-136,"points":[5098.448482163853,-993.014597103365,5124.448482163853,-
993.014597103365,5304.479347358179,-993.014597103365,5304.479347358179,-
1031.9760282560662,5484.510212552505,-1031.9760282560662,5494.510212552505,-
1031.9760282560662]},
{"from":83,"to":51,"points":[5098.448482163853,-956.629897091158,5124.448482163853,-
956.629897091158,5127.335484475555,-956.629897091158,5127.335484475555,-
968.3185488329851,5479.335484475555,-968.3185488329851,5479.335484475555,-
865.539913723238,5649.747370611477,-865.539913723238,5667.747370611477,-
865.539913723238]},
{"from":-142,"to":-151,"points":[3489.474483274612,-429.2218102713733,3489.474483274612,-
419.2218102713733,3489.474483274612,-117.18818797231938,3473.592647337112,-
117.18818797231938,3473.592647337112,184.84543432673445,3473.592647337112,210.8454343267
3445]},
{"from":35,"to":125,"points":[2988.596495608781,1379.6297308058176,2988.596495608781,1389.629
7308058176,2988.596495608781,1389.6297308058176,2940,1389.6297308058176,2940,1956,2296.82
1978859435,1956,2296.821978859435,2200.8335358149343,2296.821978859435,2210.833535814934
3]}
]}
